# Shaping polymersomes into predictable morphologies via out-of-equilibrium self-assembly

**DOI:** 10.1038/ncomms12606

**Published:** 2016-08-25

**Authors:** R. S. M. Rikken, H. Engelkamp, R. J. M. Nolte, J. C. Maan, J. C. M. van Hest, D. A. Wilson, P. C. M. Christianen

**Affiliations:** 1 Institute for Molecules and Materials, Radboud University Nijmegen, Heyendaalseweg 135, 6525 AJ Nijmegen, The Netherlands; 2 High Field Magnet Laboratory (HFML-EMFL), Radboud University Nijmegen, Toernooiveld 7, 6525 ED Nijmegen, The Netherlands

## Abstract

Polymersomes are bilayer vesicles, self-assembled from amphiphilic block copolymers. They are versatile nanocapsules with adjustable properties, such as flexibility, permeability, size and functionality. However, so far no methodological approach to control their shape exists. Here we demonstrate a mechanistically fully understood procedure to precisely control polymersome shape via an out-of-equilibrium process. Carefully selecting osmotic pressure and permeability initiates controlled deflation, resulting in transient capsule shapes, followed by reinflation of the polymersomes. The shape transformation towards stomatocytes, bowl-shaped vesicles, was probed with magnetic birefringence, permitting us to stop the process at any intermediate shape in the phase diagram. Quantitative electron microscopy analysis of the different morphologies reveals that this shape transformation proceeds via a long-predicted hysteretic deflation–inflation trajectory, which can be understood in terms of bending energy. Because of the high degree of controllability and predictability, this study provides the design rules for accessing polymersomes with all possible different shapes.

## Abstract (web summary)

Polymersomes are used in many applications, ranging from drug delivery to microfluidics, but controlling their shape is difficult. Here, Christianen and co-workers have precisely controlled polymersome shape via an out-of-equilibrium process and fully understood the mechanism of this procedure.

## Graphical abstract

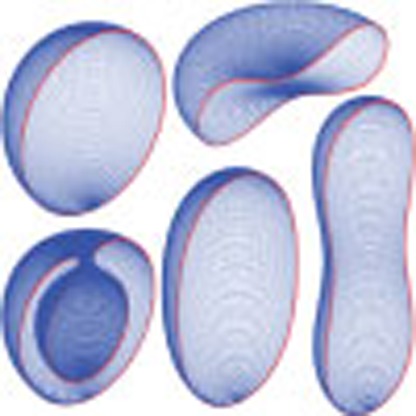

For the last 15 years, there has been an increasing interest in artificial vesicles made from amphiphilic block copolymers, often called polymersomes^1^,^2^,^3^,^4^,^5^,^6^,^7^. Polymersomes have the advantage that many of their properties are tuneable, simply by modifying the polymer-building blocks^8^,^9^. Both the hydrophilic and the hydrophobic blocks of the constituting copolymer can be adjusted to give the required surface charge, temperature sensitivity, rigidity and permeability to the vesicle. The latter two can also be tuned by adding organic solvents that plasticize the hydrophobic part of the polymersome membrane^10^,^11^. Finally, polymers can easily be functionalized with (biological) molecules, making polymersomes promising candidates for medical applications such as drug delivery^12^,^13^,^14^.

Interactions between particles such as polymersomes and cells, which are key to a range of processes such as immune regulation and cellular uptake, are strongly affected by the particle shape^15^,^16^,^17^,^18^. The shape of the polymersomes also affects their flow properties^19^, as is believed to be the case for red blood cells, biconcave disc-like erythrocytes^20^,^21^. Therefore, it is of great importance to obtain full control over the shape of polymersomes and to understand the mechanism of their interconversion. The number of studies on polymersome shape is still rather limited^22^. We showed that spherical polymersomes, assembled from poly(ethylene glycol)-polystyrene (PEG-PS), deflate into cup-shaped polymersomes called stomatocytes^23^,^24^,^25^. Rod-shaped polymersomes have been created by using crosslinkers^26^ or rehydration^27^. Bicontinuous cubic structures have been reported as well^28^. Another study showed polymersomes that deflate to form nested vesicles^29^, possibly through a sequence of intermediate vesicle shapes such as prolate spheroids, discs and stomatocytes^30^, which unfortunately were not observed. The studies above have been rather empirical in nature, despite the existence of detailed theoretical bending energy models, predicting the shape of phospholipid vesicles as a function of reduced volume and surface area difference^31^. Currently, clear and quantitative understanding of the shape transformation of polymersomes is missing, which hampers the construction of polymeric nanostructures with tailor-made shapes.

Here we report on a mechanistically fully understood new method to transform spherical PEG-PS polymersomes into a variety of shapes, such as prolate spheroids, discs and stomatocytes, which can be kinetically trapped at any stage of their formation. The polymersomes are initially formed by adding water to a molecularly dissolved polymer solution in a mixture of tetrahydrofuran (THF) and dioxane. Our approach for obtaining a controlled shape transformation is based on the notion that polymersomes are only in osmotic equilibrium at the exact moment they are formed. By adding extra water to bring the sample to a carefully chosen final solvent composition, the polymersome solution is pushed out of osmotic equilibrium. After this out-of-equilibrium self-assembly, a slow re-equilibration process starts, which leads to a predictable deflation of the spherical polymersomes into any desired polymersome morphology. The total amount of water in the solvent mixture is a crucial parameter, as it not only sets the osmotic pressure across the polymersome membrane, but, owing to the amorphous glassy nature of the PS block, it also (indirectly) modifies its flexibility and its permeability^24^. Because of the glassy nature of the PS part of the polymersome membrane combined with the fact that the PS part of the polymersome membrane is rather long (>133 repeating units), the timescales of the shape transformations are much longer (several hours^25^) compared with those observed in lipid vesicles or in polymersomes assembled from polymers with a glass transition temperature much lower than that of PS, which usually is in the order of seconds to minutes^29^,^32^. This makes it possible to probe the whole shape change process carefully in time.

The long timescale also allows us to kinetically trap the observed morphologies, including the intermediates, by quickly adding an excess of water or by a fast decrease in temperature. All the resulting shapes could be extensively characterized by *ex situ* electron microscopy, providing a quantitative mathematical description of the nanocapsules in terms of the reduced volume and surface area difference. An asymmetric deflation–inflation trajectory has only now been observed experimentally, in full accordance with the predictions of the Seifert model on liposomes^31^. Our quantitative understanding of the shape transformation within our new equilibration method paves the way for moulding polymersome nanocontainers or scaffolds in the most efficient shape, which is highly desirable for biomedical applications.

## Results

### Sample preparation

**Figure 1:**
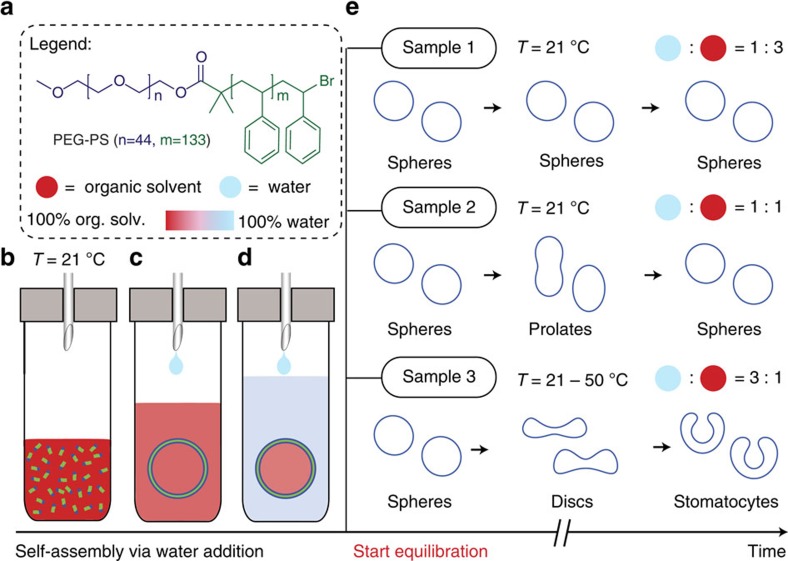
Overview of the out-of-equilibrium self-assembly approach. (**a**) Legend, showing the structure of the PEG-PS block copolymer building block and the colour scale for organic solvents, water and mixtures of the two. (**b**–**d**) Scheme of the self-assembly process. (**b**) Water is slowly (1 ml h^−1^) added to a solution of PEG-PS in THF/dioxane. (**c**) Self-assembly of spherical polymersomes occurs with identical internal and external solvent composition. (**d**) Continued addition of water to the exterior lowers the permeability to water of the polymer membrane and induces osmotic pressure, creating an out-of-equilibrium situation. (**e**) Three samples with different final water content, showing different equilibration behaviour over time (days). Sample 1 (25% water) remained spherical. Sample 2 (50% water) deflated into prolate shapes after which they inflated back to spheres. Sample 3 (75% water) deflated into discs after which they inflated into stomatocytes.

The polymersome samples were prepared as demonstrated in Fig. 1. All samples were made by first dissolving 10 mg of PEG_*n*_-PS_*m*_ (see Materials for more details) in THF:dioxane 3:2 (v/v) ranging from 1 to 3 ml (Fig. 1b). Then water was slowly added (for up to 3 h using a syringe pump) until the total volume of the mixture was 4 ml. Self-assembly of the PEG-PS into polymersomes occurred when the ratio of water:organic solvent approached 1:4 (v/v) (Fig. 1c). At the time of the self-assembly, the solvent composition in the polymersome's interior and exterior is equal. The water that is added after the polymersome formation changes the solvent composition of the surroundings, while the polymersome interior, due to the low permeability, is assumed to remain relatively unchanged. The addition of water brings the self-assembled system out of equilibrium as an osmotic pressure over the membrane is induced (Fig. 1d). The extent of osmotic pressure depends on the amount of organic solvent that is used and the amount of water that is added. The addition of extra water will also make the polymersome membrane more rigid and even less permeable to water^24^. In this manner, three different samples were prepared (Fig. 1e), varying from a sample almost in equilibrium (sample 1, 25% H_2_O) via an intermediate sample (sample 2, 50% H_2_O) to a sample far from equilibrium (sample 3, 75% H_2_O). See also Methods and Supplementary Table 1 for more details.

### Equilibration

**Figure 2:**
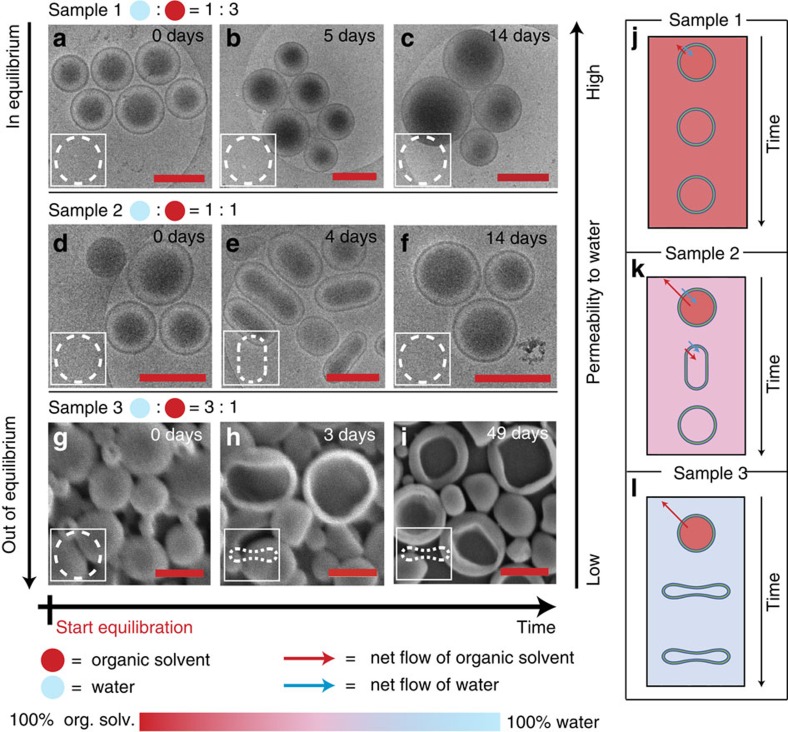
Polymersome morphology over time for different external solvent compositions. (**a**–**i**) Electron microscopy images. All scale bars are 500 nm. (**a**–**c**) Cryo-TEM images of sample 1 (25% H_2_O and 75%THF/dioxane), showing no change in morphology over time. (**d**–**f**) Cryo-TEM images of sample 2 (50%H_2_O and 50%THF/dioxane), changing from spheres to a prolate morphology after 4 days and back to spheres after 14 days. (**g**–**i**) SEM images of sample 3 (75%H_2_O and 25%THF/dioxane) changing from spheres to discs after 3 days and still remaining discs after 49 days. The left arrow indicates the direction of increasing osmotic pressure; the right arrow indicates the direction of increasing permeability to water. (**j**–**l**) Schematic explanation. (**j**) Sample 1 is near osmotic equilibrium and the most permeable to water, allowing a simultaneous exchange of water and organic solvent to alleviate the small change in osmotic pressure without changing the shape. (**k**) Sample 2 is more out of equilibrium than sample 1 and less permeable to water. Organic solvent flows out faster than water flows in, causing a small deflation to form prolates. Subsequently, the bending energy is relieved by simultaneous inflow of water and organic solvent however at a much slower rate. (**l**) Sample 3 is the most out of osmotic equilibrium and impermeable to water. Therefore, these polymersomes deflate the most to form discs, and no reinflation is possible.

Equilibration started immediately after sample preparation (Fig. 1). Each sample was studied at several points in time (days) using (cryogenic) transmission and scanning electron microscopy (TEM, cryo-TEM and SEM, see Supplementary Figs 1–3). Figure 2a–i gives an overview of those images that give the best representation of the morphology. The left arrow indicates the difference in solvent composition between the polymersome's interior and exterior, that is, how far the system deviates from osmotic equilibrium. The right arrow indicates the relative change in permeability to water while the horizontal arrow indicates the direction of time. Samples were left to stand at room temperature (21 °C) to equilibrate over time. For all three samples, three key moments that coincide with morphological changes are shown. The polymersomes of sample 1 were spherical after self-assembly and remained spherical up to 14 days (Fig. 2a–c). At intermediate days the shapes were also found to be spherical (Supplementary Fig. 1). The polymersomes of sample 2 were spherical immediately after self-assembly but started changing into prolates (spheroids and rods) after the first day, with most prolates observed at day 4 (Fig. 2d–f and Supplementary Fig. 2). However, from day 8 onwards all polymersomes were observed to have a spherical morphology again.

The polymersomes of sample 3 were spherical after self-assembly and remained spherical up until 2 days (Fig. 2g–i). After 3 days about one-third of the polymersomes had changed into discs. The same amount of discs was observed after 49 days, indicating that discs are kinetically trapped under these conditions. Magnetic birefringence (MB) measurements on sample 3 were in full agreement with these electron microscopy (EM) results (Supplementary Fig. 4). MB measures the difference in refractive index of light polarized parallel and perpendicular to a magnetic field (optical anisotropy), which is induced by magnetic alignment of structures that exhibit (dia)magnetic anisotropy^25^,^33^. When comparing structures composed of identical building blocks the MB signal is a measure of the anisotropy of the overall shape. Indeed, the MB signal increased 10-fold from the background level after 3 days, which is in accordance with the observed transition of roughly one-third of the spheres into discs.

The different shape changes of the three samples can be explained in terms of osmotic pressure and membrane permeability. Since sample 1 is close to equilibrium immediately after the self-assembly, and also the most permeable to water, the small difference in solvent composition is easily alleviated by a simultaneous outflow of organic solvent and inflow of water (Fig. 2j). The result is that the polymersomes will not deflate and therefore retain their spherical morphology. Sample 2 is more out of equilibrium than sample 1 but also less permeable to water. Therefore, after sample preparation the organic solvent flows out faster than water can flow in (Fig. 2k). This leads first to a net decrease of the inner volume and the polymersomes adopt a prolate morphology (rods), lowering the osmotic pressure at the expense of bending energy. To relieve this bending energy, the polymersomes inflate again into spheres at a speed that is limited by the inflow of water.

The polymersomes in sample 3 are the most out of equilibrium and impermeable to water. Therefore, osmotic equilibrium can only be obtained by outflow of organic solvent. Since water cannot flow back in, the polymersomes do not reinflate to spheres but retain their disc-like morphology (Fig. 2l). Because the polymersomes in sample 3 are more out of equilibrium than those in sample 2, they will deflate more, which is the reason for the different shapes, as will be explained later. Sample 3 is not observed to go back to spheres since the permeability to water at room temperature is negligible. Also, the membrane flexibility of the polymersomes of sample 3 is low since they contain the smallest amount of organic solvent to plasticize the membrane, making the timescales in which shape changes occur very large. We suspect that this relatively high rigidity is also the cause for two-thirds of the polymersomes not to undergo the shape transformation at room temperature at all.

**Figure 3:**
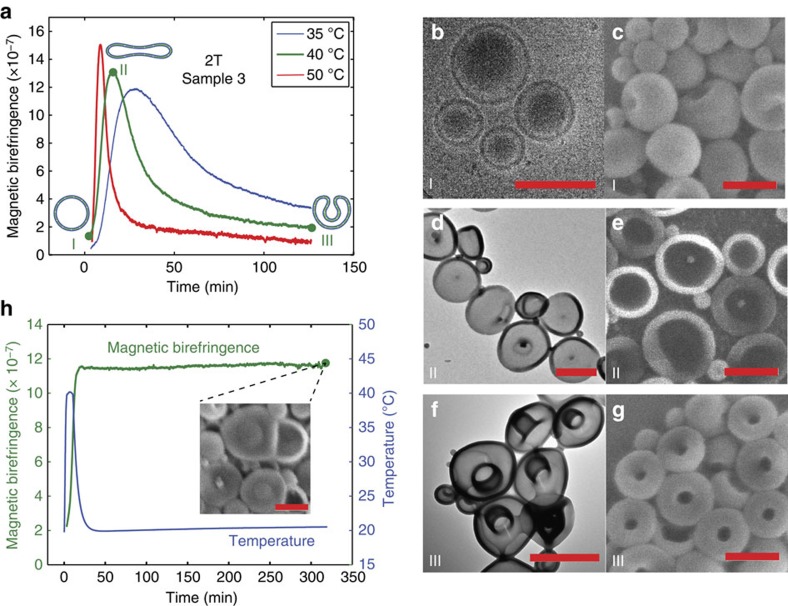
Morphology changes at elevated temperatures. (**a**) Magnetic birefringence of sample 3 (75% H_2_O and 25% THF/dioxane) as function of time at different temperatures. At each temperature, the birefringence increases to a maximum followed by a slower decrease. The higher the temperature, the faster the changes in birefringence occur. At three characteristic points on the 40 °C trace, an aliquot of the sample was injected in water to quench the structure. Cryo-TEM (**b**), TEM (**d**,**f**) and SEM (**c**,**e**,**g**) images of the quenched samples marked in **a**. For the spheres cryo-TEM was used, as spheres tend to collapse in dry TEM. At the start of the experiment the sample consists of spheres (**b**,**c**) and the birefringence is almost zero. At maximal birefringence discs are observed (**d**,**e**). At the end of the trace the sample consists of stomatocytes (**f**,**g**). (**h**) Magnetic birefringence of sample 3 as a function of time (green line), following the temperature trajectory shown in blue. By cooling the sample right before the maximal birefringence is reached the transition from discs to stomatocytes is prevented as can be seen by the constant high birefringence and the SEM picture of the sample after 320 min (inset). All scale bars are 500 nm.

To investigate the shape transformation of sample 3 at higher flexibility and permeability to water, the MB was also measured at higher temperatures; 35, 40 and 50 °C, respectively (Fig. 3a–g, Supplementary Discussions and Supplementary Figs 5 and 6). At these temperatures, the MB was observed to initially increase steeply followed by a decrease to a small, finite value. The measurements also showed that at higher temperature the changes in birefringence occurred faster and the peak value of the MB was higher. For all three measurements the shape of the polymersomes was determined by TEM, or cryo-TEM, and SEM at the beginning (I), top (II) and after the peak (III) (Fig. 3a). The shapes at these three key points were identical for all three temperatures and a representative overview for the 40 °C measurement is given in Fig. 3b–g. At the beginning of the experiment polymersomes had, as expected, a spherical morphology (Fig. 3b,c) and changed into discs at maximal birefringence (Fig. 3d,e). At the end of the experiment, the discs transformed into stomatocytes (Fig. 3f,g). There is full agreement between the EM pictures and the MB measurements. The transition from spheres to discs leads to a large increase in the MB since the polymersomes become highly anisotropic. The change from discs to stomatocytes leads to a decrease in birefringence since stomatocytes resemble more closely a sphere and are therefore much less anisotropic than discs. Interestingly, the disc morphology is maintained when quickly lowering the temperature right before the magnetic birefringence reaches a maximum, as is shown in Fig. 3h.

The temperature treatments on sample 3 demonstrate three effects. First of all, the increase in temperature leads to a much faster transition from spheres to discs. Second, the maximum birefringence at elevated temperature is about three times as high as the birefringence obtained by equilibration at room temperature (Supplementary Fig. 4). As the shapes are identical, this indicates that at elevated temperatures the number of discs becomes close to 100%, which is confirmed by EM microscopy. Finally, at elevated temperatures a further transition from discs to stomatocytes is observed, which was not observed by equilibration at room temperature. This demonstrates that, like the prolates, the disc is an intermediate morphology in the equilibration process, but that elevated temperatures are required to allow the shape change to complete. The experiments described above demonstrate how a variety of polymersome shapes can be obtained at will, by carefully setting an osmotic pressure difference during sample preparation. With magnetic birefringence and electron microscopy, the exact structure at a given moment during the shape change process can be accurately determined. All morphologies can be kinetically trapped by adding an excess of water to vitrify the polystyrene part of the membrane. Discs and stomatocytes, owing to the low concentration of organic solvent in the PS block, can also be trapped by lowering the temperature.

To investigate the reproducibility of the process, we repeated the procedures used for sample 3, using a block copolymer with a PS length of 200 units instead of 133 (sample 4). We obtained the exact same results as with sample 3 (Supplementary Figs 7 and 8), demonstrating that the procedure is reproducible, and robust against changes in the length of the PS block.

**Figure 4:**
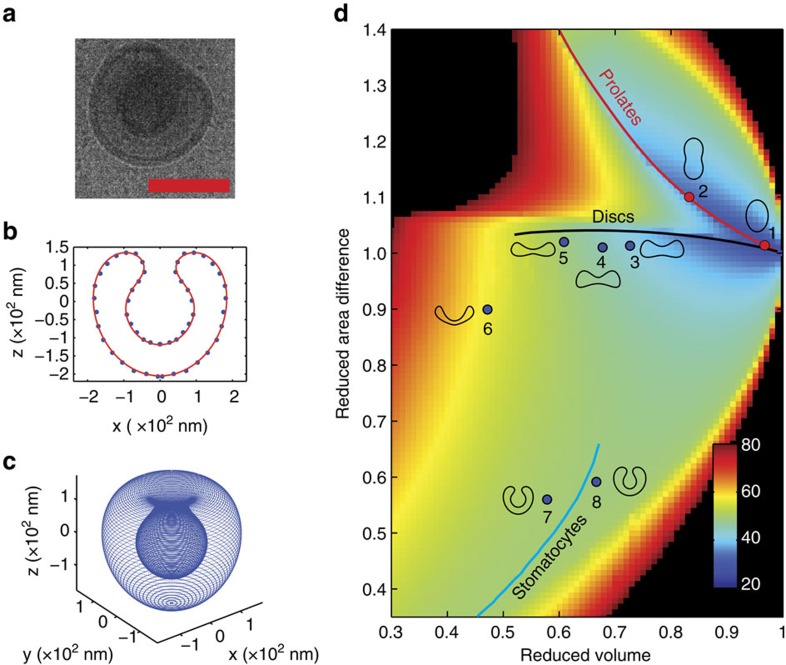
Analysis of the observed shape transitions. All observed cross-sections of the shapes that were captured with cryo-TEM and cryo-SEM were parameterized to calculate vesicle properties. (**a**) Cryo-TEM image of a stomatocyte found in sample 3 after heating to 35 °C. Scale bar, 250 nm. (**b**) Parameterization of the stomatocyte shown in **a**. Points on the membrane surface are indicated by the blue dots. The red line corresponds to the parameterization that fits the data best. (**c**) After parameterization of the cross-section, the three-dimensional structure is obtained by revolving the fit around the *z* axis since all observed structures are axisymmetric^35^. For every fitted shape, the reduced area difference and the reduced volume are calculated. (**d**) Phase diagram showing the positions of all observed shapes of sample 2 (red dots) and 3 (blue dots). The colour scale in the phase diagram indicates the minimized bending energy (*E*_bend_/*k*) for each reduced volume–reduced area difference combination. The lines drawn correspond to the calculated local minima at fixed reduced volumes for prolates (red line), discs (black line) and stomatocytes (blue line). The observed shapes are very close to the calculated local minima.

For a more quantitative description of the out-of-equilibrium shape change process, the found shapes of samples 1–3 were used to construct a phase diagram, which is presented in Fig. 4. All polymersome cross-sections obtained by cryo-TEM or cryo-SEM were fitted using a parameterization based on a Fourier series (Fig. 4a,b, Supplementary Methods, Supplementary Figs 9 and 10, and Supplementary Table 2). Since all observed shapes are cylindrically symmetric (Supplementary Methods and Supplementary Figs 11–14), their three-dimensional shape was reconstructed by revolving the fitted cross-sections around the *z* axis (Fig. 4b,c). These parameterizations were then used to calculate the reduced volume (*v*) and the reduced area difference (Δ*a*) between the outer and inner layers of the membrane (Supplementary Fig. 15). These two geometrical properties determine the position of each morphology in the phase diagram as is indicated by the red and blue dots in Fig. 4d. The reduced volume *v* quantifies the amount of deflation and reinflation, and changes only slowly for our polymersomes; Δ*a* is a free parameter we use to identify the different shapes. The colour scale in the background indicates the minimized bending energy as calculated by the spontaneous-curvature model with zero spontaneous curvature (Supplementary Methods)^31^,^34^. Trajectories of local minima as a function of *v* are indicated with solid lines. The lines corresponding to discs and stomatocytes end when there is no longer a local minimum, which occurs for discs below *v*=0.52 and for stomatocytes above *v*=0.66 (Supplementary Fig. 16)^31^,^34^. Sample 2 consists of prolates, and indeed, the shapes encountered in this sample are located very closely to the local minimum of prolates. Sample 3 consists of discs and stomatocytes, both of which are located close to the lines corresponding to the local minima of these two shapes. An intermediate between discs and stomatocytes (shape 6) was found at a reduced volume for which a disc is no longer a local minimum and a stomatocyte is lower in energy. This strongly suggests that local bending is the driving force for discs to fold into stomatocytes when the reduced volume has decreased below *v*=0.52 (Supplementary Fig. 16). Sample 2 was observed to deflate via prolates while sample 3 was observed to deflate via discs. Assuming zero spontaneous curvature, the difference in bending energy between discs and prolates is very small, in favour of prolate morphologies. It is however likely that the difference in solvent composition of the polymersome interior and exterior leads to a negative spontaneous curvature, which is larger for sample 3 than for samples 1 and 2, and which explains the preference for deflation via either prolates or discs (Supplementary Discussions).

The energy associated with osmotic pressure is up to two orders of magnitude larger than the bending energy (Supplementary Methods). Therefore, osmotic pressure will quickly be relieved through an outflow of organic solvent, causing deflation, which will lower *v*. The morphology and the corresponding Δ*a* at each value of *v* is then determined by the minimum bending energy. During this deflation, the osmotic energy will decrease but the bending energy will slightly increase. After the osmotic pressure has been alleviated, the polymersomes can reinflate by a simultaneous inflow of water and organic solvent to decrease the bending energy while maintaining osmotic balance. In case of sample 2, the polymersomes are slightly permeable to water, allowing inflow of both water and organic solvent. Indeed, inflation of prolates into spheres was observed. In case of sample 3, the permeability to water is almost absent at room temperature. Therefore, the discs are not observed to reinflate back to spheres. At elevated temperatures, the discs deflate further into stomatocytes. Once stomatocytes are formed, they reinflate as stomatocytes, but only marginally. The decrease in bending energy for inflating stomatocytes is very small compared with inflating discs or rods, which might explain why stomatocytes reinflate only marginally. However, it is striking how the deflation and inflation of sample 3 occur via different routes. One explanation for this hysteresis can be found in the spontaneous curvature model itself. One of its characteristics is that shape transitions between rods and discs and between discs and stomatocytes are discontinuous^31^,^34^,^35^,^36^,^37^, and the shapes can be trapped in local minima. Another explanation can be found in the fact that folding of a disc into a stomatocyte requires the reorganization of the individual polymers, which is accompanied by friction, which breaks down microscopic reversibility. Energetic costs associated with this reorganization can be accounted for by the release of osmotic energy during deflation. During inflation, the reorganization of the polymers might be too costly energetically, which would prevent the stomatocyte from folding back into a disc. The latter explanation is supported by the fact that elevated temperatures are required to allow sample 3 to transform all the way to stomatocytes.

In summary, we have demonstrated the manipulation of polymersomes into different shapes in a controllable manner using out-of-equilibrium self-assembly. Different shapes via different routes were observed, all by starting from the same spherical morphology. The high rigidity and small permeability of the polymersomes leads to slow kinetics, allowing to carefully monitor shape changes in time spans varying from a few hours to many days. Parameterization of the observed shapes enabled us to place all morphologies in a phase diagram and calculate their geometrical properties. The shapes obtained can be explained by a simple model based on osmotic and bending energy. The ability to vitrify the membrane by adding an excess of water makes it possible to trap all observed morphologies, including all of the intermediates. Discs can even be obtained by thermal quenching. This provides us with the possibility to prepare large batches of low-polydispersity polymersomes with well-defined and predictable shapes, such as spheres, prolates, discs and stomatocytes, at any desirable reduced volume. These differently shaped polymersomes are promising candidates for nanocontainers and scaffolds, or as building blocks for assembly of larger and more complicated architectures.

## Methods

### Materials

THF and 1,4-dioxane were purchased from Sigma-Aldrich and used as received. PEG_*n*_-PS_*m*_ was synthesized by atom-transfer radical polymerization starting from PEG-macro initiators as described previously^23^. For samples 1–3, PEG_44_-PS_133_ was used (molecular weight=16 kDa, polydispersity index=1.06). For sample 4, PEG_44_-PS_200_ was used instead (molecular weight=23 kDa, polydispersity index=1.05). The polymersomes were prepared by dissolving 10 mg of PEG_*n*_-PS_*m*_ in a 2:3 (v/v) mixture of dioxane and THF followed by the addition of H_2_O at a rate of 1 ml h^−1^ using a syringe pump, to a total sample volume of 4 ml. During the preparation the samples were stirred at 750 r.p.m. The final amount of H_2_O in the samples was 25% (sample 1), 50% (sample 2) and 75% (sample 3). The initial concentrations of PEG-PS before the addition of H_2_O was 3.33 mg ml^−1^ for sample1, 5 mg ml^−1^ for sample 2 and 10 mg ml^−1^ for sample 3. In this range, initially spherical polymersomes are formed^23^,^24^,^26^. All samples turned cloudy when the amount of H_2_O reached 20%.

The final samples were divided over HPLC vials in aliquots of 450 μl, sealed and stored at room temperature for equilibration.

### Equipment

MB was measured in a Varian V-3900 2T electromagnet using a standard polarization modulation technique^38^. A HeNe laser was used (1.5 mW, 632.8 nm) to probe the dispersion contained inside a 5 mm thick optical cell (Hellma) within a temperature-controlled environment. (Cryo-)SEM was performed on a JEOL 6,330 Cryo Field Emission Scanning Electron Microscope at an acceleration voltage of 3 kV in cryo-mode and 10 kV in dry mode. TEM was performed on a JEOL 1,010 Transmission Electron Microscope at an acceleration voltage of 60 kV. For Cryo-TEM a JEOL 2,100 cryo-Transmission Electron Microscope was used. The hydrodynamic radius was determined by Dynamic Light Scattering, performed with a Malvern Zetasizer Nano S instrument.

### Data availability

The authors declare that the data supporting the findings of this study are available within the article and its Supplementary Information or from the authors on request.

## Additional information

**How to cite this article:** Rikken, R. S. M. *et al*. Shaping polymersomes into predictable morphologies via out-of-equilibrium self-assembly. *Nat. Commun.* 7:12606 doi: 10.1038/ncomms12606 (2016).

## Supplementary Material

Supplementary Information Supplementary Figures 1-16, Supplementary Tables 1-2, Supplementary Discussion, Supplementary Methods and Supplementary References

## References

[b1] Discher B. M. . Polymersomes: tough vesicles made from diblock copolymers. Science 284, 1143–1146 (1999). 10325219 10.1126/science.284.5417.1143

[b2] Discher D. E. & Eisenberg A. Polymer vesicles. Science 297, 967–973 (2002). 12169723 10.1126/science.1074972

[b3] Opsteen J. A., Cornelissen J.J.L.M. & van Hest J. C. M. Block copolymer vesicles. Pure Appl. Chem. 76, 1309–1319 (2004).

[b4] Srinivas G., Discher D. E. & Klein M. L. Self-assembly and properties of diblock copolymers by coarse-grain molecular dynamics. Nat. Mater. 3, 638–644 (2004). 15300242 10.1038/nmat1185

[b5] Discher D. E., Bhasin N. & Johnson C. P. Covalent chemistry on distended proteins. Proc. Natl Acad. Sci. USA 103, 7533–7534 (2006). 16682625 10.1073/pnas.0602388103 PMC1472479

[b6] LoPresti C., Lomas H., Massignani M., Smart T. & Battaglia G. Polymersomes: nature inspired nanometer sized compartments. J. Mater. Chem. 19, 3576–3590 (2009).

[b7] Mai Y. Y. & Eisenberg A. Self-assembly of block copolymers. Chem. Soc. Rev. 41, 5969–5985 (2012). 22776960 10.1039/c2cs35115c

[b8] Le Meins J. F., Sandre O. & Lecommandoux S. Recent trends in the tuning of polymersomes' membrane properties. Eur. Phys. J. E 34, 1–17 (2011). 10.1140/epje/i2011-11014-y 21337017

[b9] Rodriguez-Garcia R. . Polymersomes: smart vesicles of tunable rigidity and permeability. Soft Matter 7, 1532–1542 (2011).

[b10] Holder S. J. & Sommerdijk N.A.J.M. New micellar morphologies from amphiphilic block copolymers: disks, toroids and bicontinuous micelles. Polym. Chem. 2, 1018–1028 (2011).

[b11] Yu Y. S. & Eisenberg A. Control of morphology through polymer-solvent interactions in crew-cut aggregates of amphiphilic block copolymers. J. Am. Chem. Soc. 119, 8383–8384 (1997).

[b12] van Dongen S. F. M. . A block copolymer for functionalisation of polymersome surfaces. Macromol. Rapid Commun. 29, 321–325 (2008).

[b13] Brinkhuis R. P., Rutjes F.P.J.T. & van Hest J. C. M. Polymeric vesicles in biomedical applications. Polym. Chem. 2, 1449–1462 (2011).

[b14] Debets M. F. . Nanobody-functionalized polymersomes for tumor-vessel targeting. Macromol. Biosci. 13, 938–945 (2013). 23695978 10.1002/mabi.201300039

[b15] Balmert S. C. & Little S. R. Biomimetic delivery with micro- and nanoparticles. Adv. Mater. 24, 3757–3778 (2012). 22528985 10.1002/adma.201200224 PMC3627374

[b16] Kolhar P. . Using shape effects to target antibody-coated nanoparticles to lung and brain endothelium. Proc. Natl Acad. Sci. USA 110, 10753–10758 (2013). 23754411 10.1073/pnas.1308345110 PMC3696781

[b17] Petros R. A. & DeSimone J. M. Strategies in the design of nanoparticles for therapeutic applications. Nat. Rev. Drug Discov. 9, 615–627 (2010). 20616808 10.1038/nrd2591

[b18] Perry J. L., Herlihy K. P., Napier M. E. & DeSimone J. M. PRINT: a novel platform toward shape and size specific nanoparticle theranostics. Acc. Chem. Res. 44, 990–998 (2011). 21809808 10.1021/ar2000315 PMC4157651

[b19] Rothenbuhler J. R., Huang J.-R., DiDonna B. A., Levine A. J. & Mason T. G. Mesoscale structure of diffusion-limited aggregates of colloidal rods and disks. Soft Matter 5, 3639–3645 (2009).

[b20] Uzoigwe C. The human erythrocyte has developed the biconcave disc shape to optimise the flow properties of the blood in the large vessels. Med. Hypotheses 67, 1159–1163 (2006). 16797867 10.1016/j.mehy.2004.11.047

[b21] Doshi N., Zahr A. S., Bhaskar S., Lahann J. & Mitragotri S. Red blood cell-mimicking synthetic biomaterial particles. Proc. Natl Acad. Sci. USA 106, 21495–21499 (2009). 20018694 10.1073/pnas.0907127106 PMC2799852

[b22] Chang H. Y., Sheng Y. J. & Tsao H. K. Structural and mechanical characteristics of polymersomes. Soft Matter 10, 6373–6381 (2014). 25062328 10.1039/c4sm01092b

[b23] Kim K. T. . Polymersome stomatocytes: controlled shape transformation in polymer vesicles. J. Am. Chem. Soc. 132, 12522–12524 (2010). 20718470 10.1021/ja104154t

[b24] Meeuwissen S. A., Kim K. T., Chen Y., Pochan D. J. & van Hest J. C. M. Controlled shape transformation of polymersome stomatocytes. Angew. Chem. Int. Ed. 50, 7070–7073 (2011). 10.1002/anie.201102167 21688372

[b25] Rikken R. S. M. . Probing morphological changes in polymersomes with magnetic birefringence. Chem. Commun. 50, 5394–5396 (2014). 10.1039/c3cc47483f 24212531

[b26] van Oers M. C. M., Rutjes F.P.J.T. & van Hest J. C. M. Tubular polymersomes: a cross-linker-induced shape transformation. J. Am. Chem. Soc. 135, 16308–16311 (2013). 24156517 10.1021/ja408754z

[b27] Robertson J. D. . pH-sensitive tubular polymersomes: formation and applications in cellular delivery. ACS Nano 8, 4650–4661 (2014). 24724711 10.1021/nn5004088

[b28] La Y. . Colloidal inverse bicontinuous cubic membranes of block copolymers with tunable surface functional groups. Nat. Chem. 6, 534–541 (2014). 24848240 10.1038/nchem.1946

[b29] Salva R. . Polymersome shape transformation at the nanoscale. ACS Nano 7, 9298–9311 (2013). 24047230 10.1021/nn4039589

[b30] Hubert D. H. W. . Morphology transformations of DODAB vesicles reminiscent of endocytosis and vesicular traffic. Langmuir 16, 8973–8979 (2000).

[b31] Seifert U., Berndl K. & Lipowsky R. Shape transformations of vesicles: phase diagram for spontaneous- curvature and bilayer-coupling models. Phys. Rev. A 44, 1182–1202 (1991). 9906067 10.1103/physreva.44.1182

[b32] Lipowsky R. The morphology of lipid-membranes. Curr. Opin. Struct. Biol. 5, 531–540 (1995). 8528770 10.1016/0959-440x(95)80040-9

[b33] van Rhee P. G. . Polymersome magneto-valves for reversible capture and release of nanoparticles. Nat. Commun. 5, 5010 (2014). 25248402 10.1038/ncomms6010 PMC4176683

[b34] Seifert U. Configurations of fluid membranes and vesicles. Adv. Phys. 46, 13–137 (1997).

[b35] Heinrich V., Brumen M., Heinrich R., Svetina S. & Žekš B. Nearly spherical vesicle shapes calculated by use of spherical harmonics: axisymmetric and nonaxisymmetric shapes and their stability. J. Phys. II France 2, 1081–1108 (1992).

[b36] Seifert U. & Lipowsky R. in Handbook of Biological Physics Vol. 1, Elsevier (1995).

[b37] Jarić M., Seifert U., Wintz W. & Wortis M. Vesicular instabilities: the prolate-to-oblate transition and other shape instabilities of fluid bilayer membranes. Phys. Rev. E 52, 6623–6634 (1995). 10.1103/physreve.52.6623 9964179

[b38] Maret G. & Dransfeld K. in Strong and Ultrastrong Magnetic Fields and Their Applications Vol. 57, ed. Herlach F. 143–204Springer (1985).

