# Supplementary material for: Shaping polymersomes into predictable morphologies via out-of-equilibrium self-assembly
Source: Nat Commun. 2016 Aug 25;7:12606. doi: 10.1038/ncomms12606 (PMC5007325; doi:10.1038/ncomms12606)
Supplement: Supplementary Information — Supplementary Figures 1-16, Supplementary Tables 1-2, Supplementary Discussion, Supplementary Methods and Supplementary References [file ncomms12606-s1.pdf]

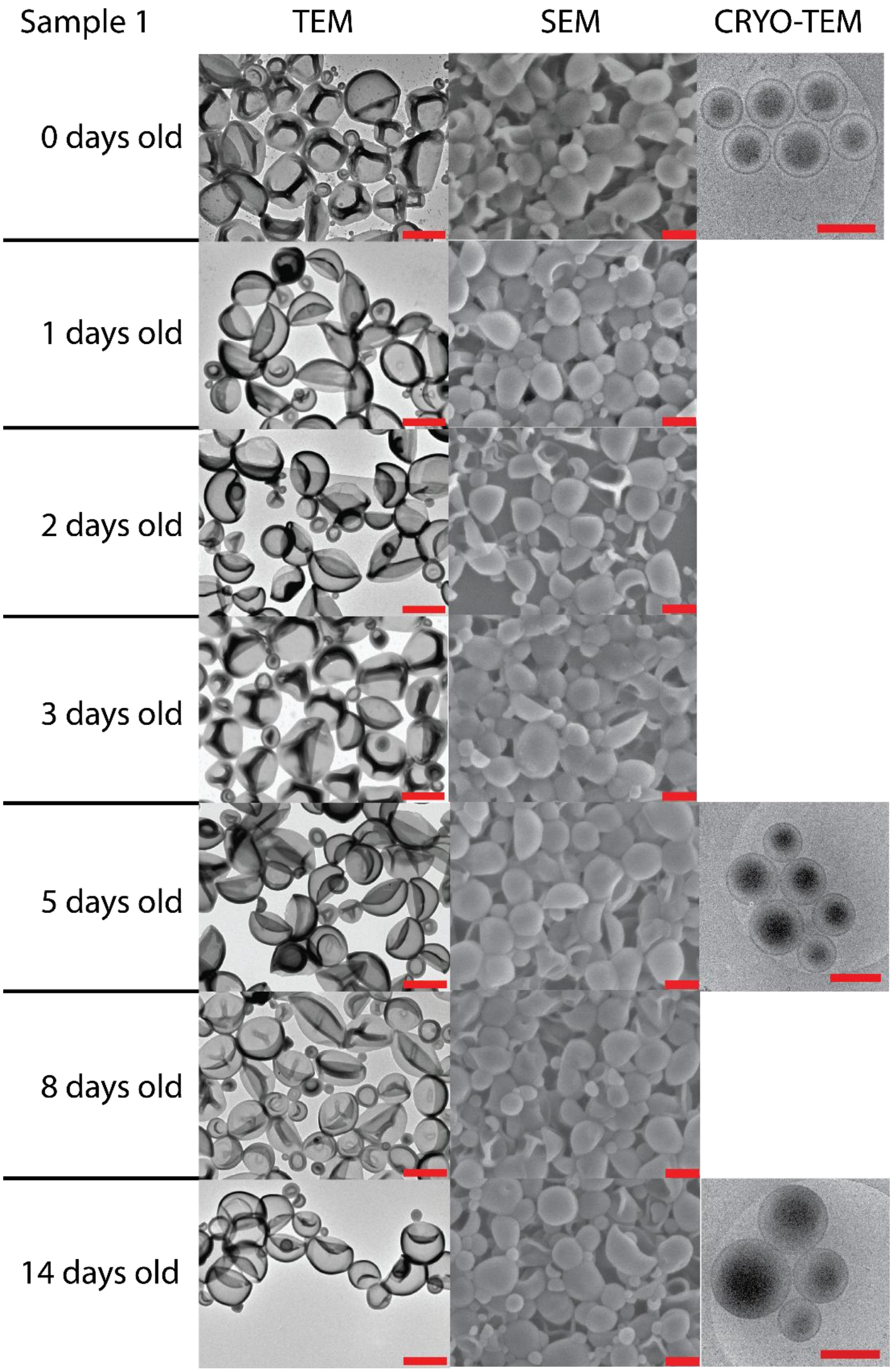

Supplementary Figure 1. TEM and SEM images of sample 1 at seven different days after self-assembly. Cryo-TEM (right) clearly shows that the morphology remains spherical over time. The indented structures, due to drying, in normal TEM (left) can still be identified as spheres. All scale bars are 500 nm.

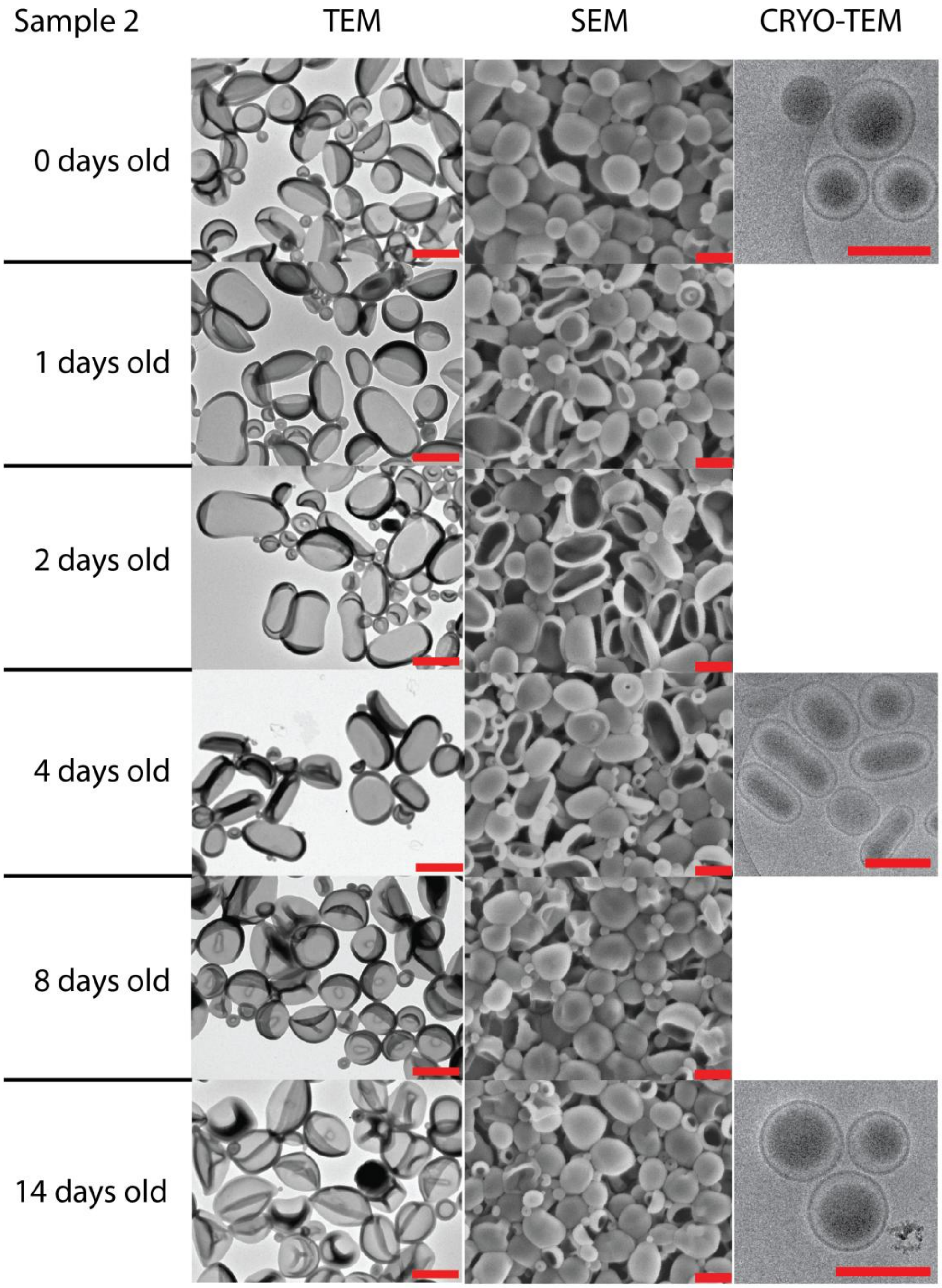

Supplementary Figure 2. TEM and SEM images of sample 2 at six different days after self-assembly. Cryo-TEM is shown on the right. As can be seen, the morphology changed from spherical to prolate after 1 day with the number of prolates increasing when the sample is 3 and 4 days old. After 8 and 14 days only spheres were observed. All scale bars are 500 nm.

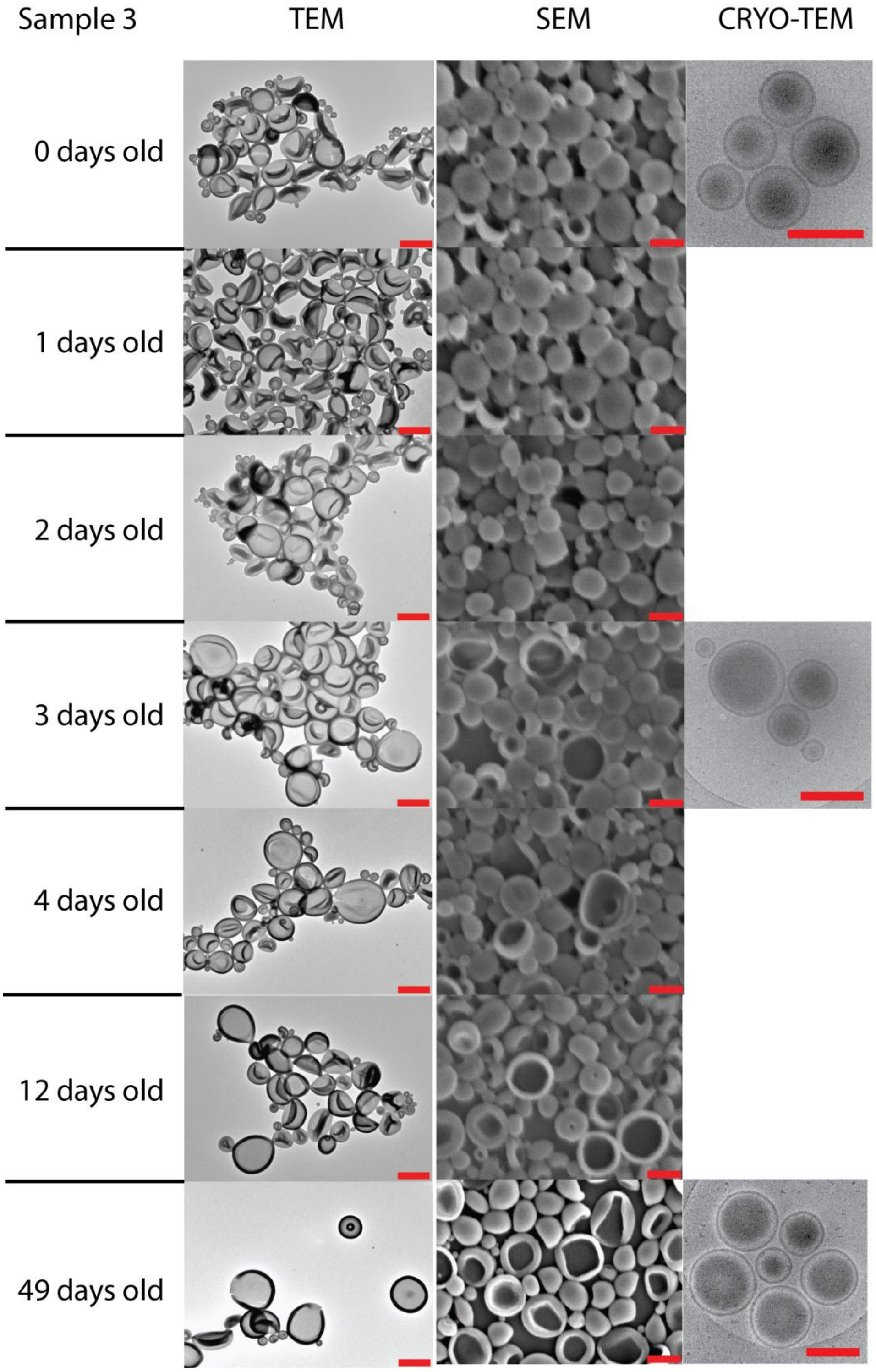

Supplementary Figure 3. TEM and SEM images of sample 3 at seven different days after self-assembly. Cryo-TEM is shown on the right. As can be seen, the morphology changed from spherical to disc with the number of discs increasing over time. All scale bars are 500 nm.

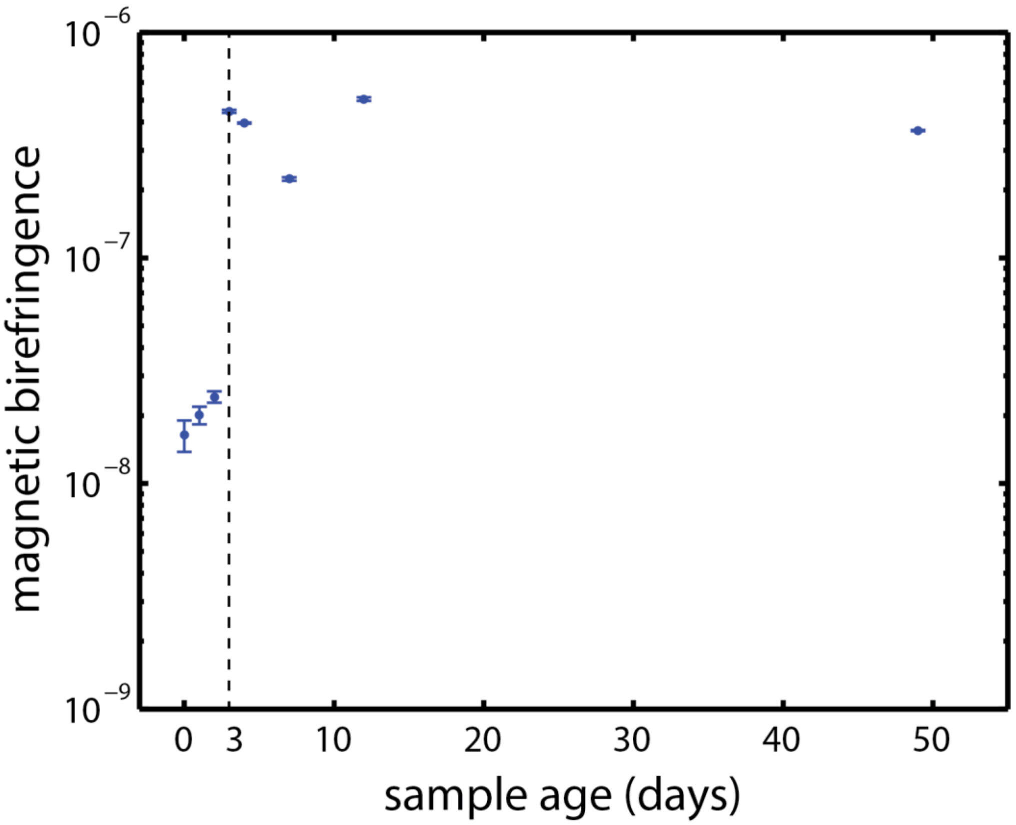

Supplementary Figure 4. Magnetic birefringence of sample 3 as function of sample age. The magnetic birefringence of sample 3 was measured at room temperature over multiple days to follow the equilibration process. A sudden increase of an order of magnitude in birefringence was observed when the sample was 3 days old. These findings are in complete accordance with the EM images which show a significant increase in discs after this time.

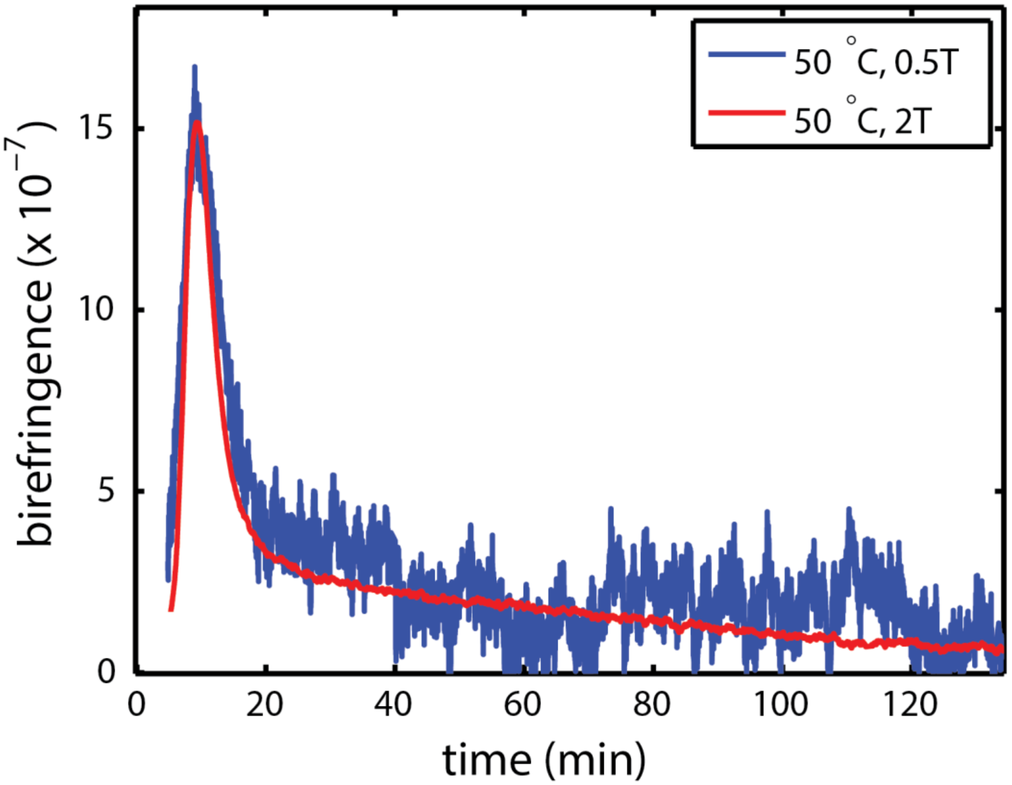

Supplementary Figure 5. Magnetic field dependence of sample 3 at 50 ^{0}C. The magnetic birefringence of sample 3 at 50 ^{0}C measured once at 0.5 T and once at 2 T. For comparison the amplitude of the 0.5 T measurement is scaled to a 2 T measurement by multiplication with 16, since the magnetic birefringence was observed to scale with B^2 up to 2 T. The two curves overlap indicating that the magnetic field does not accelerate or decelerate the rate at which the changes in morphology occur. Therefore a 2 T magnetic field can be used for probing sample 3

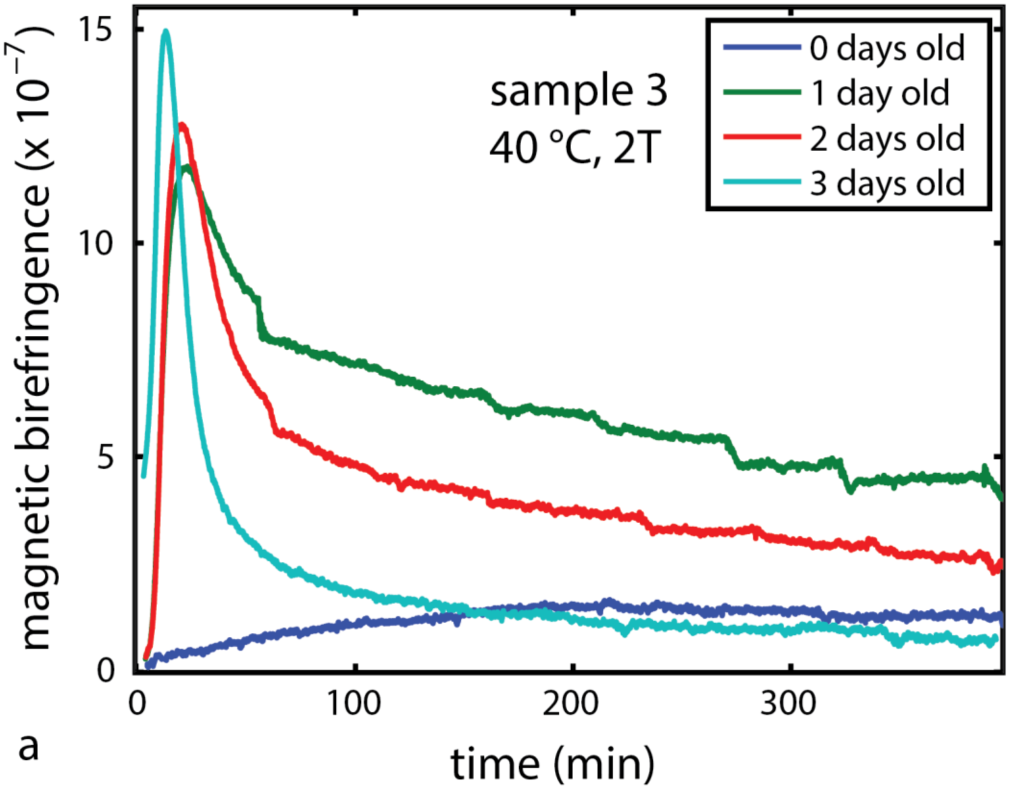

Supplementary Figure 6. Magnetic birefringence of sample 3 at elevated temperatures. Magnetic birefringence of sample 3 measured at 40 ^{0}C at 0, 1, 2 and 3 days after self-assembly. As can be seen there is almost no change in birefringence when the sample is just self-assembled (0 days old). The older the sample gets, the more easily the sample can make the transition to discs and subsequently stomatocytes. The whole transition becomes smooth and stable with temperature when the magnetic birefringence at t = 0 is at least 3% of the maximal birefringence (at the peak).

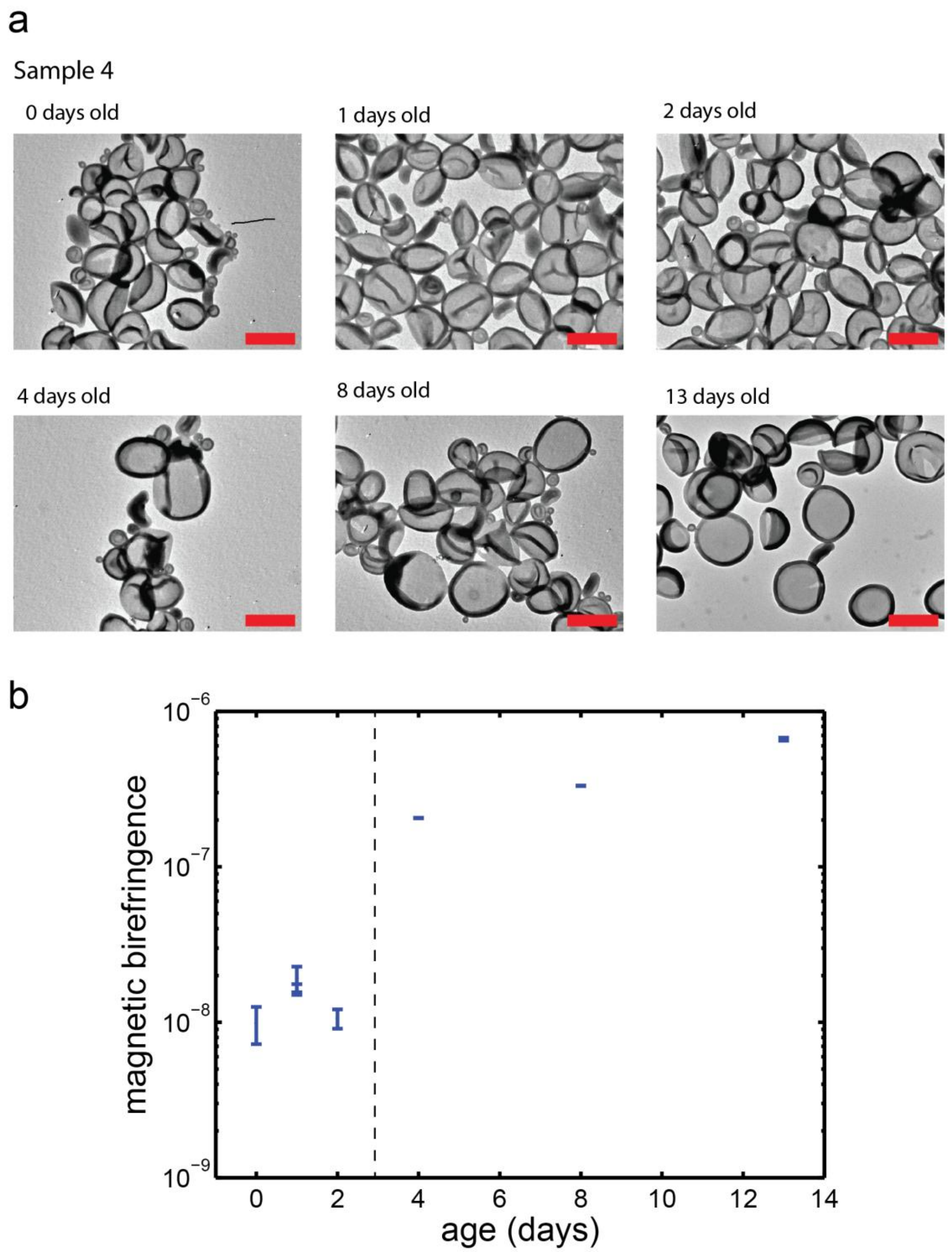

Supplementary Figure 7. Results of the equilibration experiments on sample 4. The sample was made from PEG_{44}-PS_{200} rather than PEG_{44}-PS_{133} while the self-assembly procedure was identical to that of sample 3. (a) In the TEM images we see that the first discs are observed when the sample was 4 days old. (b) The magnetic birefringence measurements are in agreement with the results from TEM. The observed effects and trends are very similar to those observed for sample 3 demonstrating that the experiment is quite robust to changes in the length of the PS block. All scale bars are 500 nm.

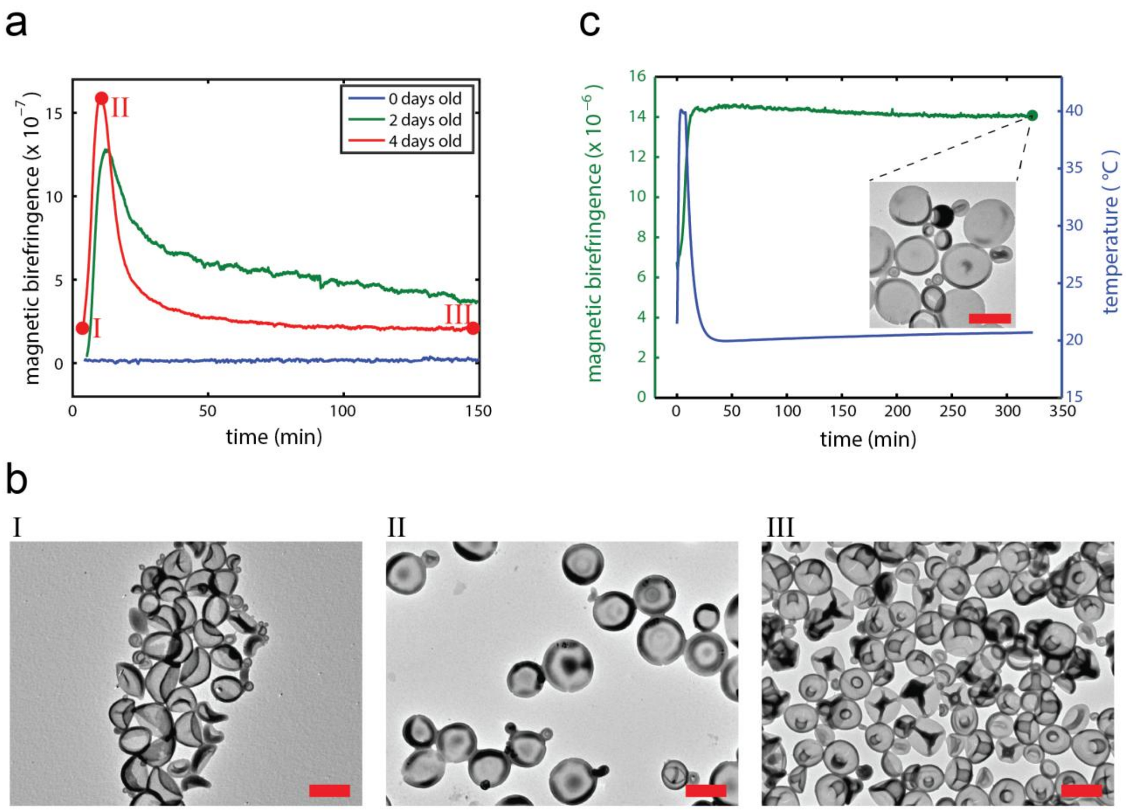

Supplementary Figure 8. Overview of the results from the measurements on sample 4 at elevated temperatures. The sample was made from PEG_{44}-PS_{200} rather than PEG_{44}-PS_{133} while the self-assembly procedure was identical to that of sample 3. (a) The magnetic birefringence measurements at 40 ºC show the same trend as sample 3 when measured over time. (b) TEM images are in agreement with the changes in magnetic birefringence. At point I the morphology is spherical (collapsed by drying), while point II shows discs and point III shows stomatocytes. (c) Also for sample 4 it was possible to thermally quench the discs by lowering the temperature right before the magnetic birefringence reached a maximum. These trends are all identical to the ones observed for sample 3. All scalebars are 500 nm.

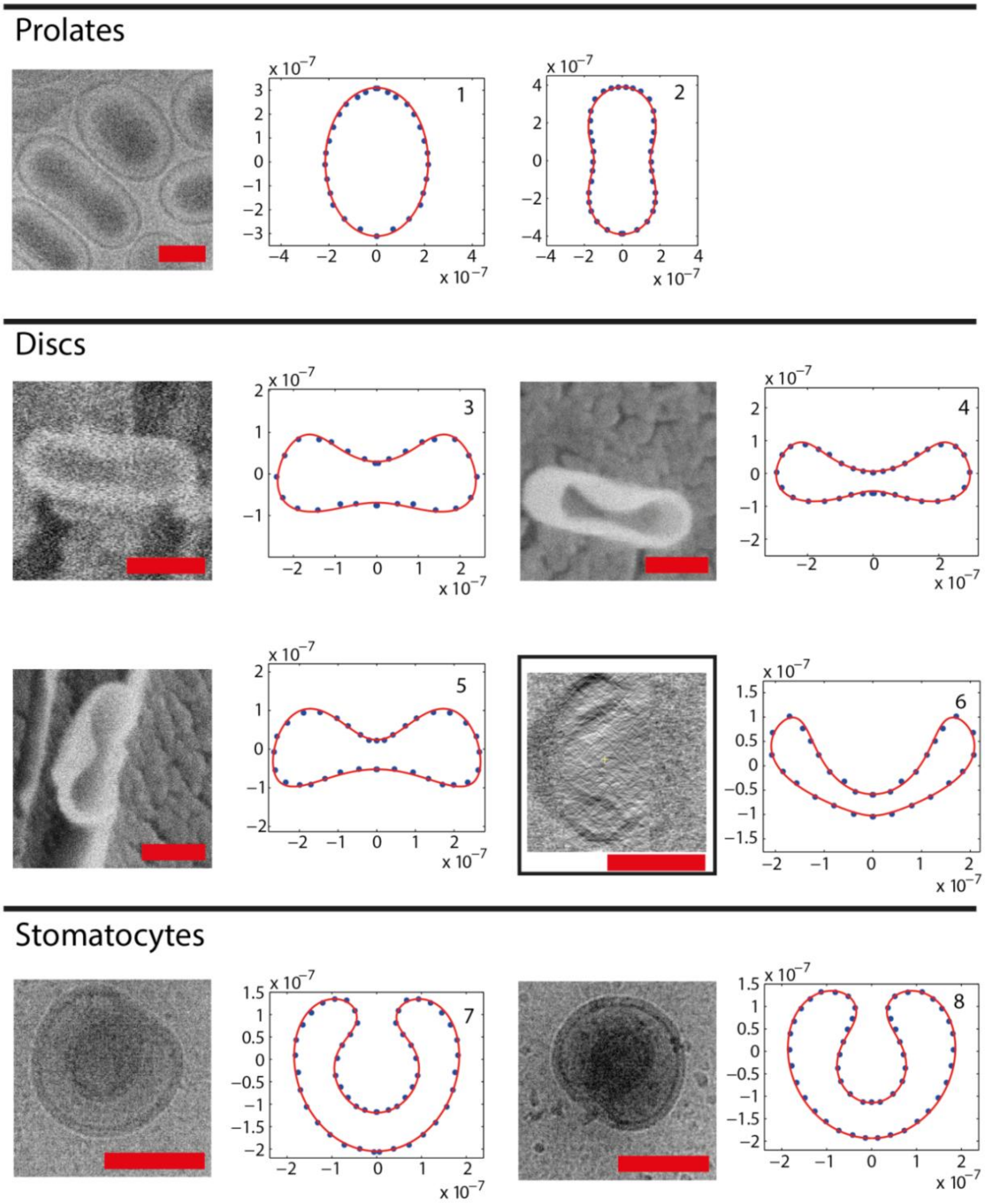

Supplementary Figure 9. Parameterization of different shapes from EM images. The cryo-TEM image of the prolates was taken from sample 2 which was 4 days old. All EM images of the discs were taken on sample 3 which was warmed to 35 ^{0}C and quenched when the magnetic birefringence was at a maximum. All discs were imaged with cryo-SEM, except for the fourth which was a reconstruction from a cryo-TEM tomogram. The discontinuity in the background is an artefact of the reconstruction. The cryo-TEM images of the stomatocytes were taken from sample 3 which was warmed to 35 ^{0}C and quenched at the end of the magnetic birefringence curve. For all EM-images, one half of the cross section was fitted since the structures are cylindrically symmetric. For the cryo-SEM images, those halves were used that were parallel to the surface. All scale bars are 250 nm.

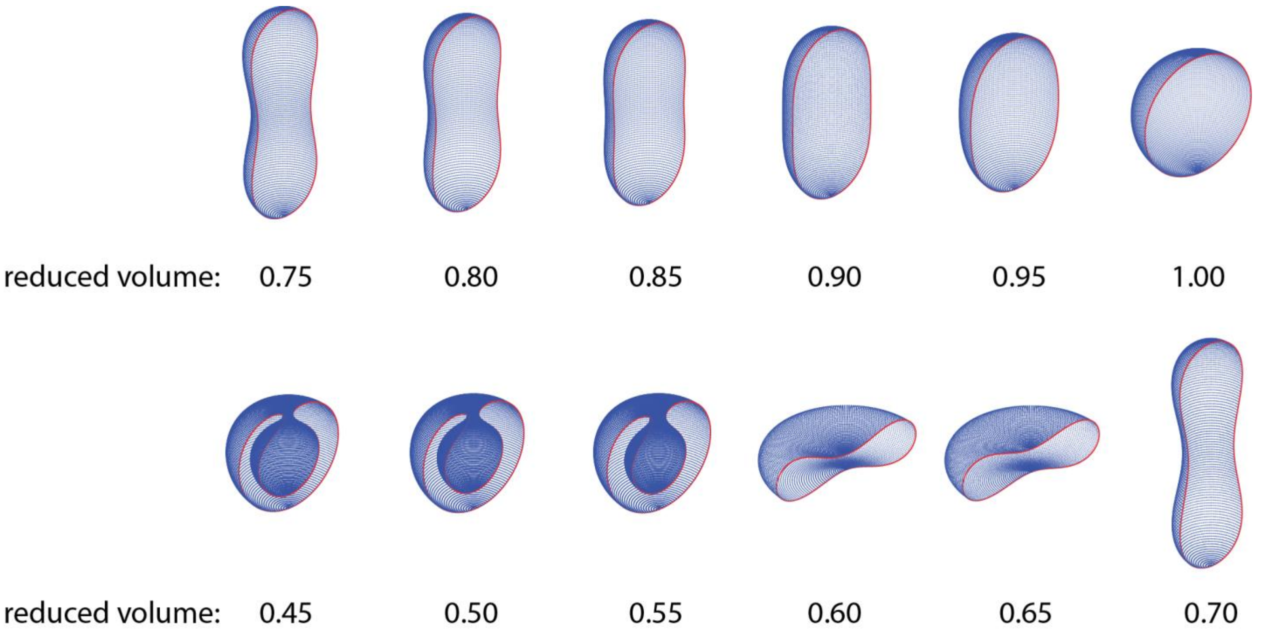

Supplementary Figure 10. Calculated shapes of lowest bending energy as function of the reduced volume *v*. Using the spontaneous curvature model with $c_0 = 0$, the shapes of lowest energy were calculated using the parameterization given in Supplementary Information 3. The calculated shapes are in full agreement with those found by Seifert *et al.*[6], demonstrating that it is sufficient to only use the first four terms in our parameterization (Supplementary equations 1-5).

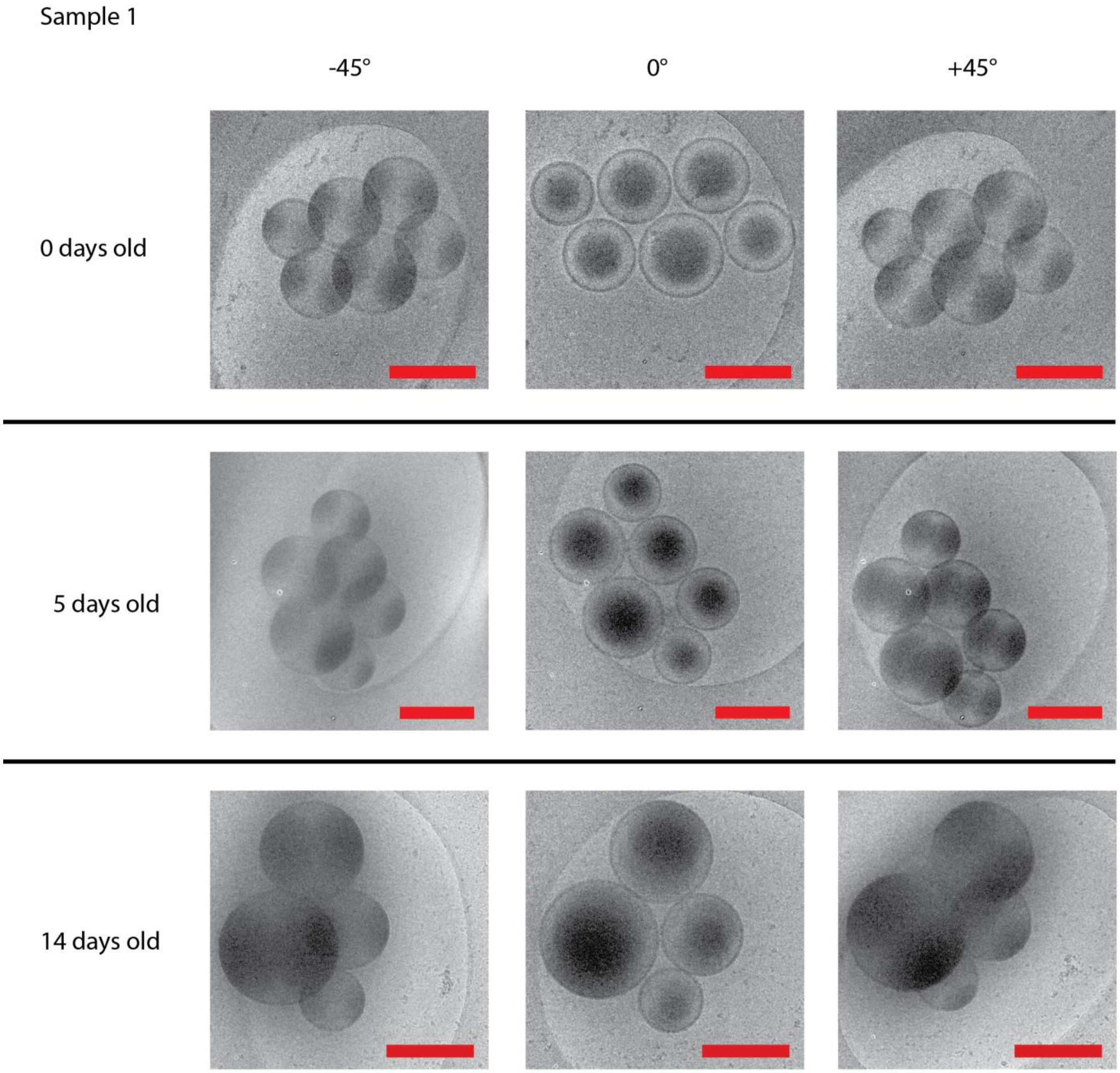

Supplementary Figure 11. Angle-dependent cryo-TEM on sample 1. The sample at day 0, 5 and 14 are shown under 3 different angles: -45^{0}, 0^{0} and 45^{0}. At all days, we see circular structures under all angles, proving that the 3D shape of the vesicles remains spherical over time. All scale bars are 500 nm.

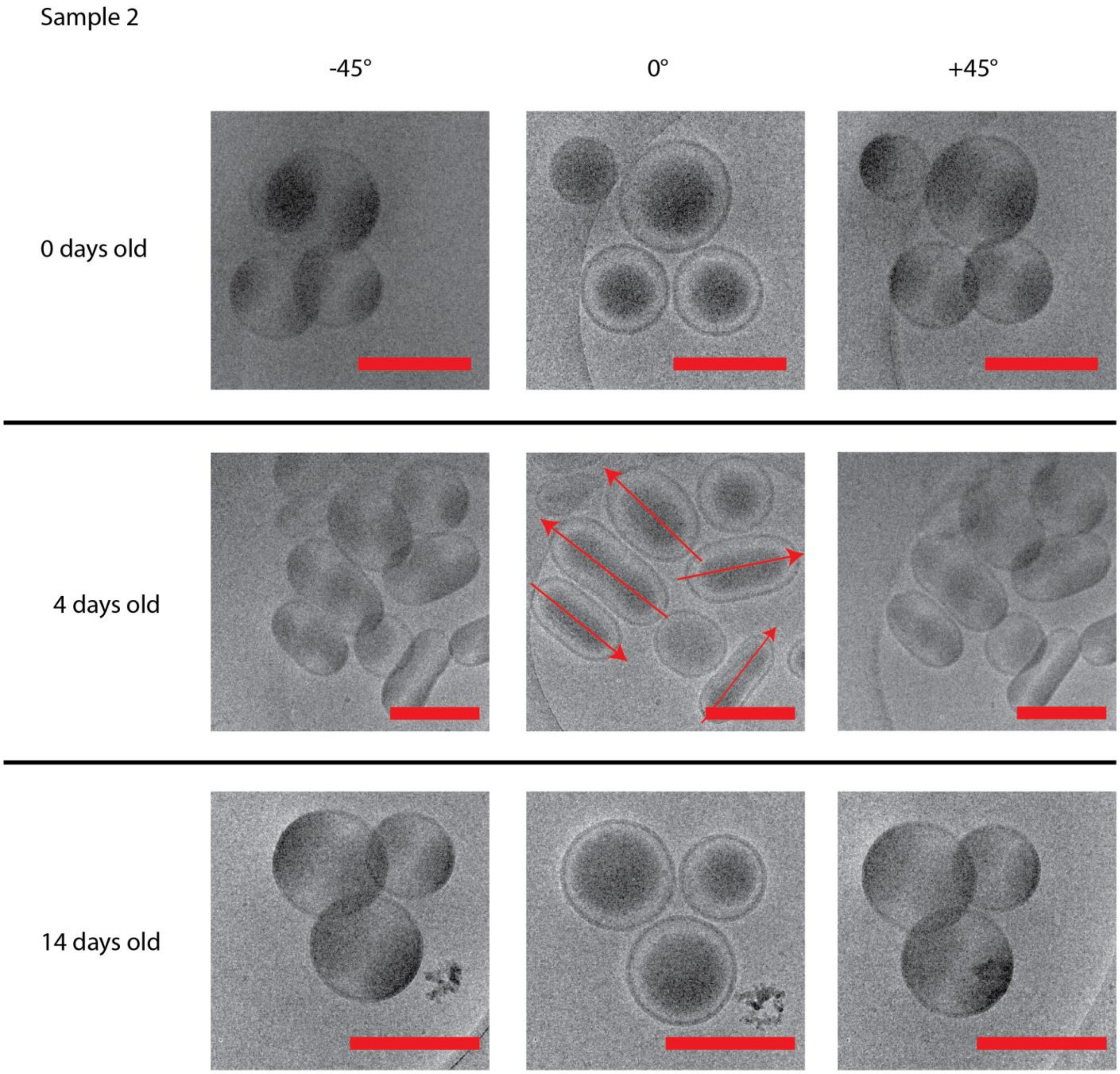

Supplementary Figure 12. Angle-dependent cryo-TEM on sample 2. The sample at day 0, 4 and 14 are shown under 3 different angles: -45^0, 0^0 and 45^0. At day 0 and 14 we see circular structures under all angles, proving that the 3D shape of the vesicles at these times is indeed spherical. At day 4 we see the prolates. The images recorded at -45^0 and 45^0 look similar and show a shorter aspect ratio. The largest aspect ratio was observed at 0^0, suggesting that the prolates are lying with their symmetry axis in-plane as indicated by the red arrows. All scale bars are 500 nm.

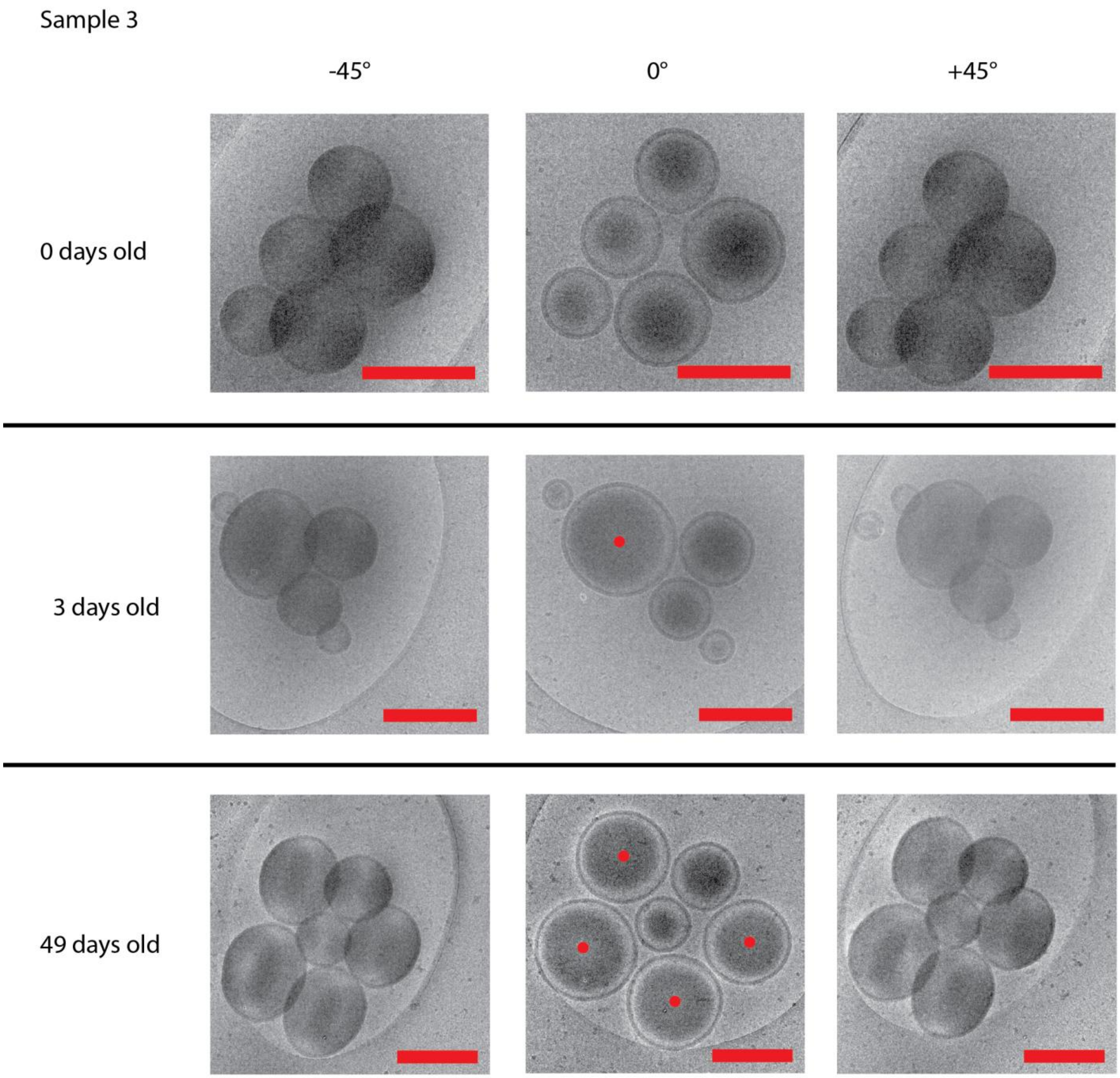

Supplementary Figure 13. Angle-dependent cryo-TEM on sample 3. The sample at day 0, 3 and 49 are shown under 3 different angles: -45^0, 0^0 and 45^0. At day 0 we see circular structures under all angles, proving that the 3D shape of the vesicles at this time is indeed spherical. At day 3 and 49 we see that there are also structures which look dented and not circular when looked at from -45^0 or 45^0. These disc-shapes polymersomes are lying with their symmetry axis out-of-plane, since they are only circular when viewed from the top (0^0). Some structures remain spherical however as can be seen by their circular representation at all different angles. For the discs, the symmetry axis are indicated by the red dots (out-of-plane arrow). All scale bars are 500 nm.

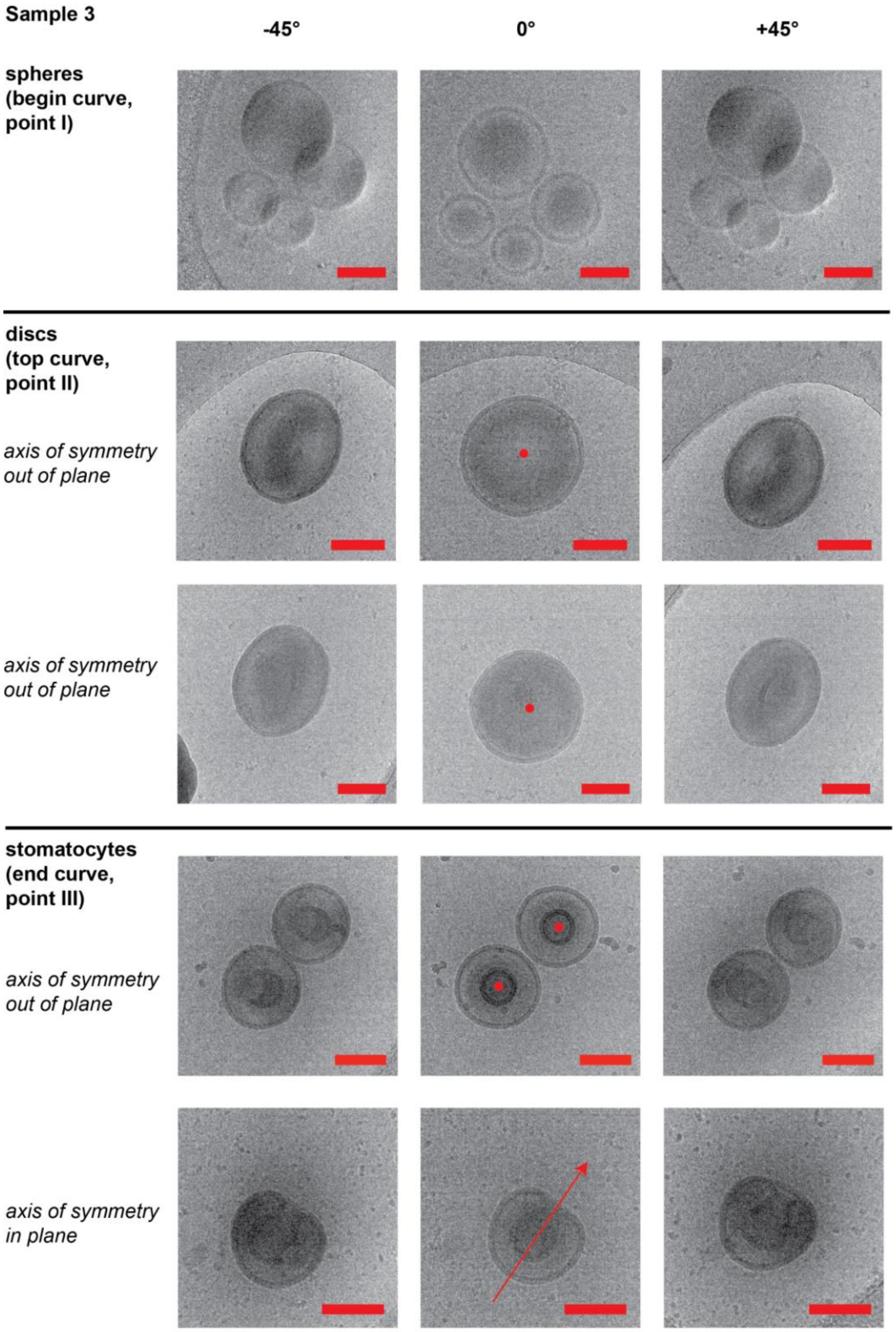

Supplementary Figure 14. Angle-dependent cryo-TEM on sample 3 at elevated temperatures (35-40 ^{0}C). Before heating (point I in MB curve of figure 3), the shape is spherical. At point II of the MB curve in figure 3, only discs were observed. The symmetry axis is out-of-plane (indicated with red dot) since the discs looks circular when imaged at 0°. The stomatocytes observed at the end of the experiment (point III in the MB curve of figure 3) are found either with their symmetry axis out-of-plane (red dot) or with their symmetry axis in-plane (red arrow). Those found with their symmetry axis in-plane can be used for parameterization. All scale bars are 500 nm.

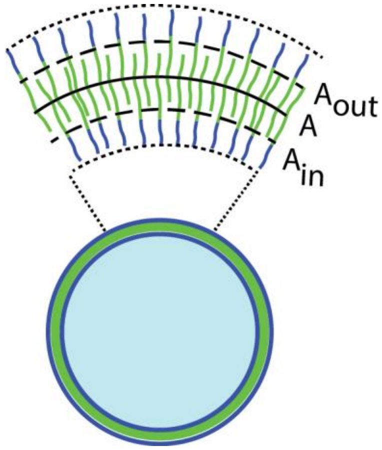

Supplementary Figure 15. Area difference of a polymersome. A vesicle of a particular shape has three surface areas. The actual surface area A is defined by the midplane of the bilayer (solid line). The outer and inner surface areas (A_{out} and A_{in}) are determined by the midplanes of the outer and inner monolayer respectively (dashed lines). The reduced area difference is calculated by dividing the area difference ($A_{out} – A_{in}$) of a vesicle by that of a sphere with the same surface area A. Likewise, the reduced volume is calculated by dividing the volume of the vesicle by that of a sphere.

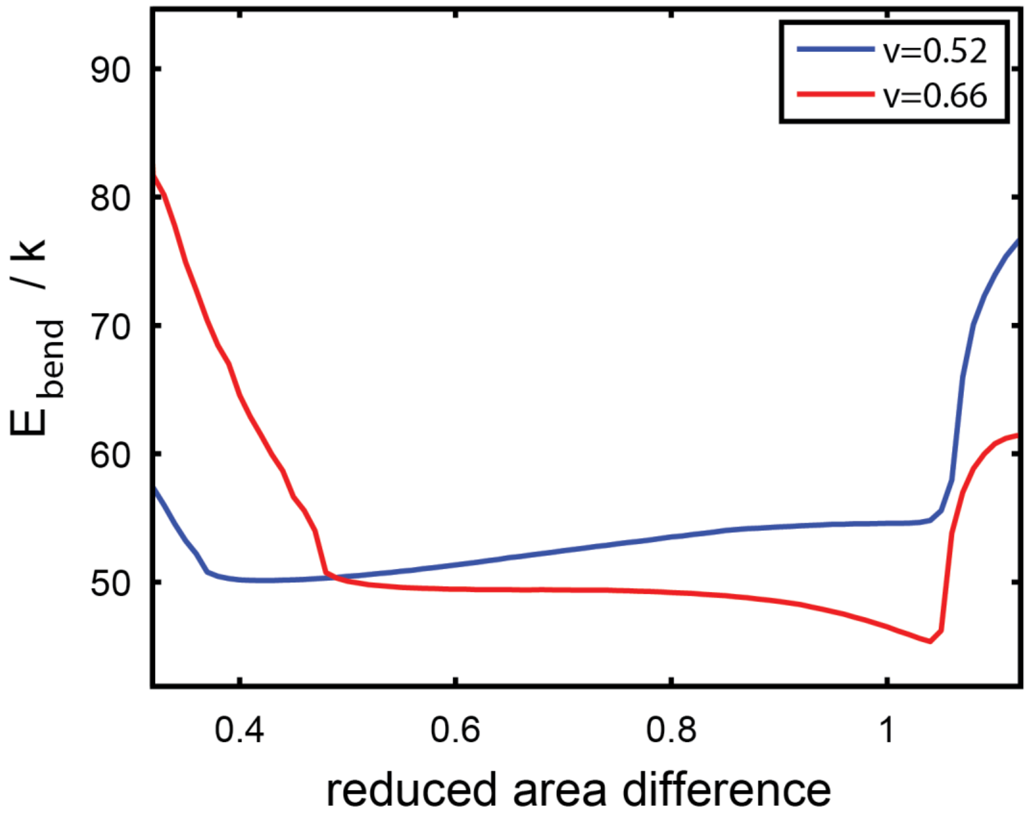

Supplementary Figure 16. Minimized bending energy as function of reduced area difference for a reduced volume of 0.52 (blue) and 0.66 (red). At v = 0.52 (and lower) the disc is no longer a local minimum, but the stomatocyte is (at a reduced area difference of 0.42). At v = 0.66 (and higher) the stomatocyte shape is no longer in a local minimum but the disc is (at a reduced area difference of 1.04).

Supplementary Table 1. Properties of the samples used directly after preparation. Hydrodynamic radius (R_h) of samples and their PDI and spread were determined on a quenched sample (injected in an excess of water).

Sample	Average hydro-dynamic radius and spread (nm)	PDI	% (v/v) water	% (v/v) THF	% (v/v) 1,4 dioxane
Sample 1 for equilibration at room temperature	272 ± 84	0.093	25	45	30
Sample 2 for equilibration at room temperature	265 ± 96	0.130	50	30	20
Sample 2 for temperature measurements	275 ± 86	0.096	50	30	20
Sample 3 for equilibration at room temperature	223 ± 56	0.062	75	15	10
Sample 3 for temperature measurements	231 ± 62	0.072	75	15	10
Sample 3 for testing magnetic field effect	227 ± 79	0.121	75	15	10
Sample 4 for equilibration at room temperature	231 ± 72	0.095	75	15	10

Supplementary Table 2. Parameters obtained from the fits shown in Supportive Fig. 9.

Shape	a_1 ($\cdot 10^{-7}$)	a_2 ($\cdot 10^{-7}$)	a_3 ($\cdot 10^{-7}$)	a_4 ($\cdot 10^{-7}$)	b_1 ($\cdot 10^{-7}$)	b_2 ($\cdot 10^{-7}$)	b_3 ($\cdot 10^{-7}$)	b_4 ($\cdot 10^{-7}$)	v	Δa
1	2.15	0	0	0	3.1	0	0	0	0.9672	1.0139
2	1.995	0	0.50625	0	3.4	0	0.5	0	0.8323	1.1003
3	2.25	-0.15	-0.075	0.075	0.882	-0.195	-0.39	0	0.7266	1.0132
4	2.76	0	-0.15	0.15	0.75	-0.23625	-0.45	0	0.6085	1.0200
5	2.55	-0.315	-0.075	0	0.882	-0.125	-0.5	-0.015	0.6776	1.0102
6	1.936	-0.055	-0.05775	0.165	0.495	-0.77	-0.275	-0.033	0.4718	0.8993
7	1.1925	-0.4275	0.72	-0.07442	0.009	-1.35	0.42	-0.27	0.5784	0.5597
8	1.1	-0.53	0.7	-0.072	-0.0252	-1.32	0.42	-0.22	0.6666	0.5914

Supplementary Discussions

Magnetic birefringence

To prove that the magnetic field of 2 T did not influence the experiment by inducing magnetic deformation[1,2] or by changing the rate at which the shape changes occur, the MB measurement at 50 °C was repeated at 0.5 T for comparison (Supplementary Fig. 5). No difference in amplitude or transition rate was observed between the two, indicating that a magnetic field of 2 T can be used to probe polymersome morphologies in sample 3.

MB of sample 3 at elevated temperature as function of time

The MB shown in Fig 3a. was measured when sample 3 was 1 day old. One can also repeat this temperature experiment at different days after self-assembly when equilibration is in a different stage. Temperature measurements on sample 3 were therefore also performed at different days after self-assembly (Supplementary Fig. 6). It shows that the sample needs to equilibrate a day before the transition becomes possible. A complete smooth transition becomes possible when the initial birefringence is at least $3.5 \cdot 10^{-8}$ which is typically after 1 to 3 days. The most probable explanation for this delay is the fact that in the preparation of sample 3 the water content is increased to 75%. This large amount of water is expected to initially vitrify the polystyrene part of the membrane, thereby blocking any shape changes. Over time, organic solvent trapped in the lumen of the polymersomes diffuses into the membrane and plasticises it sufficiently for shape changes to occur. Indeed, the addition of a large excess of water (>95%) is a standard method to permanently quench PEG-PS polymersomes by vitrification of the PS part of the membrane[3-5].

Effect of solvent composition on deflation

To understand why spheres deflate via prolates in sample 2 and via discs in sample 3 one should take into account that the solvent composition in the polymersome interior is different from the exterior after sample preparation. Since the interior contains a larger amount of organic solvent than the exterior, one would expect the membrane to be more swollen on the polymersome interior than on the exterior. In the spontaneous curvature model this would lead to the introduction of a negative spontaneous curvature which is largest for sample 3 and smallest for sample 1. For a reduced spontaneous curvature between 0 and -1.18, the prolates are still lowest in energy when deflating from a sphere. For $c_0 < -1.18$ the discs will be lower in energy when deflating from a sphere.[7] This is indeed what we experimentally observe. Sample 3 indeed deflates via discs while sample 2 deflates via prolates.

Supplementary Methods

Parameterization of shapes and calculations

For each different shape, we selected electron microscopy images such that the axis of symmetry was as closely as possible in the plane of the image. For rods and stomatocytes the clearest pictures were obtained in cryo-TEM and we could readily find good cross sections. Discs always lie flat in cryo-TEM with their axis of symmetry out-of-plane. For those, freeze-fractioning cryo-SEM was required as with this technique all orientations are present. We could only observe the cross section of the intermediate shape between disc and stomatocyte with cryo-TEM tomography. Using a Matlab script we extracted key points (see Supplementary Figure 9) from the cross sections that subsequently were subjected to a fitting routine.

All parameterizations of shapes are performed using the following fitting formula:

$$x = \sum_{n=1}^{l} a_n \sin(n \cdot v) \quad (1)$$

$$z = \sum_{n=1}^{l} b_n \cos(n \cdot v) \quad (2)$$

With v from 0 to π. The three dimensional shape is obtained by revolving the fit around the z-axis.

$$x = \left(\sum_{n=1}^{l} a_n \sin(n \cdot v)\right) \cdot \cos(u) \quad (3)$$

$$y = \left(\sum_{n=1}^{l} a_n \sin(n \cdot v)\right) \cdot \sin(u) \quad (4)$$

$$z = \sum_{n=1}^{l} b_n \cos(n \cdot v) \quad (5)$$

With u from 0 to 2π.

To find out an acceptable number of l, we reproduced the shapes of minimal bending energy as was calculated before by Seifert *et al.* for different reduced volume.[6,7] For $l = 4$, the exact same solutions could be reproduced (Supportive Fig. 9), demonstrating that it is sufficient to only use the first 4 terms in Supplementary equations 1-5.

Fittings and calculations are performed with a home-made Matlab script in which the sum of the squares of the errors between points on the membrane and the fit is minimalized.

The surface area A of a closed parameterized shape can be calculated by:

$$A = \int_{u=0}^{2\pi} \int_{v=0}^{\pi} J(v,u) \cdot dv \cdot du \quad (6)$$

with $J(v,u)$, the Jacobian, defined as:

$$J(v,u) = \left| \frac{\partial \mathbf{r}}{\partial v} \times \frac{\partial \mathbf{r}}{\partial u} \right| \quad (7)$$

with $\mathbf{r} = (x, y, z)$, the vector describing a certain point with coordinates x, y and z on the vesicle surface. The volume V of a closed parameterized shape can be calculated by:

$$V = \int_{u=0}^{2\pi} \int_{v=0}^{\pi} z(v) \cdot \left(\frac{\partial x}{\partial u} \frac{\partial y}{\partial v} - \frac{\partial y}{\partial u} \frac{\partial x}{\partial v} \right) \cdot dv \cdot du \tag{8}$$

Working out Supplementary equation 6 for our parameterization gives:

$$A = 2\pi \int_{v=0}^{\pi} J(v) \cdot dv \tag{9}$$

with:

$$J(v) = \begin{pmatrix} [\sum_{n=1}^{4} n \cdot b_n \sin(n \cdot v) \cdot \sum_{n=1}^{4} a_n \sin(n \cdot v)]^2 \\ +[\sum_{n=1}^{4} n \cdot a_n \cos(n \cdot v) \cdot \sum_{n=1}^{4} a_n \sin(n \cdot v)]^2 \end{pmatrix}^{0.5} \tag{10}$$

Working out Supplementary equation 8 for our parameterization gives:

$$V = 2\pi \int_{v=0}^{\pi} z(v) \cdot (\sum_{n=1}^{4} a_n \sin(n \cdot v))(\sum_{n=1}^{4} n \cdot a_n \cos(n \cdot v))\, dv \tag{11}$$

The reduced volume, v, is then defined as:

$$v = V / \left(\frac{4}{3} \pi \left(\frac{A}{4\pi} \right)^{1.5} \right) \tag{12}$$

The area difference of a vesicle can be expressed by:

$$\Delta A = 4d \oint H dA \tag{13}$$

With H the mean curvature which can be written in terms of the parameterization[8], d is the distance between the midplanes of the whole membrane and the midplane of one monolayer, which corresponds to ¼th of the membrane thickness

The reduced area difference, Δa, is then defined as:

$$\Delta a = \Delta A / \left(16\pi d \sqrt{\frac{A}{4\pi}} \right) \tag{14}$$

Determining the symmetry axis of the vesicles

To determine the symmetry axis of every shape encountered, we recorded cryo-TEM images of all shapes at 3 different angles to obtain more information about their 3D shape and direction of symmetry axis. For the equilibration experiments at room temperature of samples 1, 2 and 3, these images are shown in Supplementary Figs 11, 12 and 13 respectively. For the temperature experiments on sample 3, the cryo-TEM images are shown in figure 14.

It was observed that rods always lie with their symmetery axis in-plane, as is shown in Supplementary Figure 12. Discs lie with their symmetry axis out-of-plane (Supplementary Figure 13). To parameterize a shape, its symmetry axis must lie in-plane. Therefore, we used cryo-SEM to obtain the cross-sections of some discs instead. This was done by breaking a frozen disc sample in two and only look for those discs that lie perpendicular in the ice and that are broken in half to show its cross section. The cry-TEM images do show that the discs are cylinderically symmetric, so the symmetry axis can be drawn parallel to the plane of symmetry of the cross-section found in SEM.

When performing cryo-TEM, the stomatocytes were found to lie either with their symmetry axis in-plane or out-of-plane as can be seen in Supplementary figure 14. The ones that lie in-plane can be paramerized. The stomatocytes that lie with their symmetry axis out-of-plane do show that the stomatocytes are completely cylinderically symmetric.

Energetic calculations

The total energy of a polymersome vesicle can be described as the sum of the osmotic energy, E_{os}, the bending energy, E_{bend}:[6,7]

$$E_{tot} = E_{os} + E_{bend} \tag{15}$$

The osmotic energy is expressed as:

$$E_{os} = R_g T \left[n \cdot \ln\left(\frac{V}{V_0}\right) - c(V - V_0) \right] \tag{16}$$

With R_g the gas constant, T the absolute temperature, n the number of moles of solute inside the vesicle, V the volume of the vesicle lumen after equilibration, V_o the volume of the vesicle lumen before equilibration and c the concentration of the solute.

The bending energy can be expressed as:

$$E_{bend} = \frac{\kappa}{2} \oint (2H - C_0)^2 dA \tag{17}$$

With κ the bending constant, H the mean curvature, C_0 the spontaneous curvature and A the surface area.

Osmotic pressure. The concentrations of solvents range between 1.17 M (for dioxane in sample 3) and 41.67 M (for water in sample 3). The concentration differences are therefore in the order of ten mole. When taking a vesicle of radius 250 nm (which is average), the osmotic energy is in the order of 10^{-16} J.

Bending energy. Depending on the flexibility of the polymersome membrane, which also depends on the amount of organic solvent present, the bending constant is usually in the order of 10-100 kT with longer polymer chains even higher than 100 kT.[9,10] Since the bending energy of a sphere is $4\pi\kappa$, the bending energy of polymersomes is in the order of 10^{-18} J. So there is 2 orders of magnitude difference between the bending energy and the osmotic energy.

Since E_{os} is around two orders of magnitudes larger than E_{bend}, one can assume that shape changes will not occur if they induce a large osmotic pressure difference.

Supplementary References

1. Van Rhee, P.G. *et al.* Polymersome magneto-valves for reversible capture and release of nanoparticles. *Nature Commun.* **5**, 5010 (2014).
2. Shklyarevskiy, I.O. *et al.* Magnetic deformation of self-assembled sexithiophene spherical nanocapsules. *J. Am. Chem. Soc.* **127**, 1112-1113 (2005).
3. Chang, H.-Y., Sheng, Y.-J. & Tsao, H.-K. Structural and mechanical characteristics of polymersomes. *Soft Matter* **10**, 6373-6381 (2014).
4. Kim, K.-T. *et al.* Polymersome stomatocytes: controlled shape transformation in polymer vesicles. *J. Am. Chem. Soc.* **132**, 12522-12524 (2010).
5. Meeuwissen, S.A., Kim, K.-T., Chen, Y., Pochan, D.J. & van Hest, J.C.M. Controlled shape transformation of polymersome stomatocytes. *Angew. Chem. Int. Ed.* **50**, 7070-7073 (2011).
6. Seifert, U., Berndl, K. & Lipowsky, R. Shape transformations of vesicles: phase diagram for spontaneous curvature and bilayer-coupling models. *Phys. Rev. A* **44**, 1182-1202 (1991).
7. Seifert, U. Configurations of fluid membranes and vesicles. *Adv. Phys.* **46**, 13-137 (1997).
8. Wolfram website. Link: http://mathworld.wolfram.com/MeanCurvature.html. Accessed September 2014.
9. Bermúdez, H., Hammer, D.A. & Discher, D.E. Effect of bilayer thickness on membrane bending rigidity. *Langmuir* **20**, 540-543 (2004).
10. Rodriguez-Garcia, e*t al.* Polymersomes: smart vesicles of tunable rigidity and permeability. *Soft Matter* **7**, 1532-1542 (2011).
